# Differential role of pre- and postsynaptic neurons in the activity-dependent control of synaptic strengths across dendrites

**DOI:** 10.1371/journal.pbio.2006223

**Published:** 2019-06-05

**Authors:** Mathieu Letellier, Florian Levet, Olivier Thoumine, Yukiko Goda

**Affiliations:** 1 RIKEN Brain Science Institute, Wako, Saitama, Japan; 2 Interdisciplinary Institute for Neuroscience, University of Bordeaux, Bordeaux, France; 3 Interdisciplinary Institute for Neuroscience, Centre National de la Recherche Scientifique (CNRS) UMR 5297, Bordeaux, France; 4 Bordeaux Imaging Center, University of Bordeaux, Bordeaux, France; 5 Bordeaux Imaging Center, CNRS UMS 3420, Bordeaux, France; 6 Bordeaux Imaging Center, INSERM US04, Bordeaux, France; 7 RIKEN Center for Brain Science, Wako, Saitama, Japan; ICM - Institut du Cerveau et de la Moelle épinière, France

## Abstract

Neurons receive a large number of active synaptic inputs from their many presynaptic partners across their dendritic tree. However, little is known about how the strengths of individual synapses are controlled in balance with other synapses to effectively encode information while maintaining network homeostasis. This is in part due to the difficulty in assessing the activity of individual synapses with identified afferent and efferent connections for a synapse population in the brain. Here, to gain insights into the basic cellular rules that drive the activity-dependent spatial distribution of pre- and postsynaptic strengths across incoming axons and dendrites, we combine patch-clamp recordings with live-cell imaging of hippocampal pyramidal neurons in dissociated cultures and organotypic slices. Under basal conditions, both pre- and postsynaptic strengths cluster on single dendritic branches according to the identity of the presynaptic neurons, thus highlighting the ability of single dendritic branches to exhibit input specificity. Stimulating a single presynaptic neuron induces input-specific and dendritic branchwise spatial clustering of presynaptic strengths, which accompanies a widespread multiplicative scaling of postsynaptic strengths in dissociated cultures and heterosynaptic plasticity at distant synapses in organotypic slices. Our study provides evidence for a potential homeostatic mechanism by which the rapid changes in global or distant postsynaptic strengths compensate for input-specific presynaptic plasticity.

## Introduction

Synapses are highly diverse in their morphology, molecular composition, and efficacy [1–4], even for the inputs and outputs of single neurons [5–8]. Such synapse heterogeneity in single neurons may be the consequence of differences in particular afferent or efferent connection types, represent some inherent cell-specific feature, or both. For instance, along the axon of cortical layer 2/3 pyramidal neurons, the boutons have presynaptic strengths that differ according to the target neuron type [9]; along the dendritic tree of hippocampal CA1 neurons, synaptic strengths are specific to the afferent connection type [10] and can vary also according to the distance from the soma [11–13].

Beyond the variability of individual synapses that are set by connection types and intrinsic cell-specific properties, synaptic strengths undergo dynamic changes over time. Long-lasting forms of activity-dependent Hebbian synaptic plasticity such as long-term potentiation (LTP), which in the hippocampus is believed to represent a major mechanism for encoding episodic memories [14–16], introduce an additional complexity to the synapse diversity. The observed heterogeneity of synaptic strengths could represent a snapshot of the capacity or the state of information storage [17–20]. Notably, induction of Hebbian plasticity at a given synapse can influence neighboring synapses and bias the outcome of subsequent plasticity induction [21,22], it can produce heterosynaptic plasticity at inactive synapses [23–25], and it has been postulated to trigger compensatory, homeostatic forms of synaptic plasticity [26–30]. How the interrelated changes in synaptic strengths are regulated across a defined synapse population for efficient learning remains to be clarified [31,32].

Mapping the dynamic spatial distribution of synaptic strengths across dendrites has provided some insights into the coordinated regulation of synapses embedded in the same network [17,33]. In an emerging view, synapses that carry related information undergo similar synaptic strength changes, which result in spatially clustered presynaptic [34,35] and postsynaptic strengths [22,36–39]. The synchronous activation of nearby synapses triggers local dendritic activity to enable a nonlinear dendritic branchwise integration of information [40–42]. By binding inputs carrying related information independently of other inputs carrying distinct information, such dendritic nonlinearity is thought to enhance the computational capacity of neurons [31,32,43]. In some neurons, however, instead of being clustered, related inputs are found distributed across the dendritic tree [44], a spatial organization that favors linear integration for implementing highly synapse-specific learning [45].

Whether synapse-specific or branch-specific, learning-induced synaptic strength changes can influence neighboring synapses by promoting the spread of plasticity-related factors that reduce the threshold to subsequent plasticity-inducing activity [46]. Learning-induced synaptic plasticity also triggers heterosynaptic compensatory changes [47–49] that could result from competition for resources between stimulated and nonstimulated synapses [30,50,51]. Despite extensive efforts to delineate how particular types of input activity alters the efficacy and connectivity of synapses, the logic underlying the spatial organization of individual synaptic strengths and their dynamic changes are not well understood, especially with respect to the pre- and the postsynaptic compartments of each synapse [1,52]. Such analyses are hampered by the difficulties in assessing the strengths of individual synapses in brain tissue with dense connectivity and in identifying the specific connections to which the individual synapses belong [1,53]. Furthermore, whereas the sizes of dendritic spines are readily visualized and serve as a proxy for postsynaptic strengths [8,10,34,54], axonal bouton volumes or the total synaptic vesicle pool sizes are poorly correlated to presynaptic strengths [55,56]. Therefore, ready measures of individual presynaptic strengths in intact tissue preparations are scarce.

In this study, by combining multiple patch-clamp recordings with fluorescence live imaging of hippocampal neurons in primary cultures and organotypic slices, we have sought to characterize the basic features of cellular rules controlling the activity-dependent spatial distribution of individual synaptic strengths across the incoming axons and dendrites. We also aimed to determine whether compensatory changes might be imposed as a requisite mechanism to maintain network homeostasis [26,57–59]. Our findings support a potential homeostatic mechanism by which rapid changes in global or distant postsynaptic strengths compensate for input-specific presynaptic plasticity.

## Results

### Presynaptic strengths are determined by both the presynaptic cell identity and the postsynaptic dendritic branch

**Fig 1:**
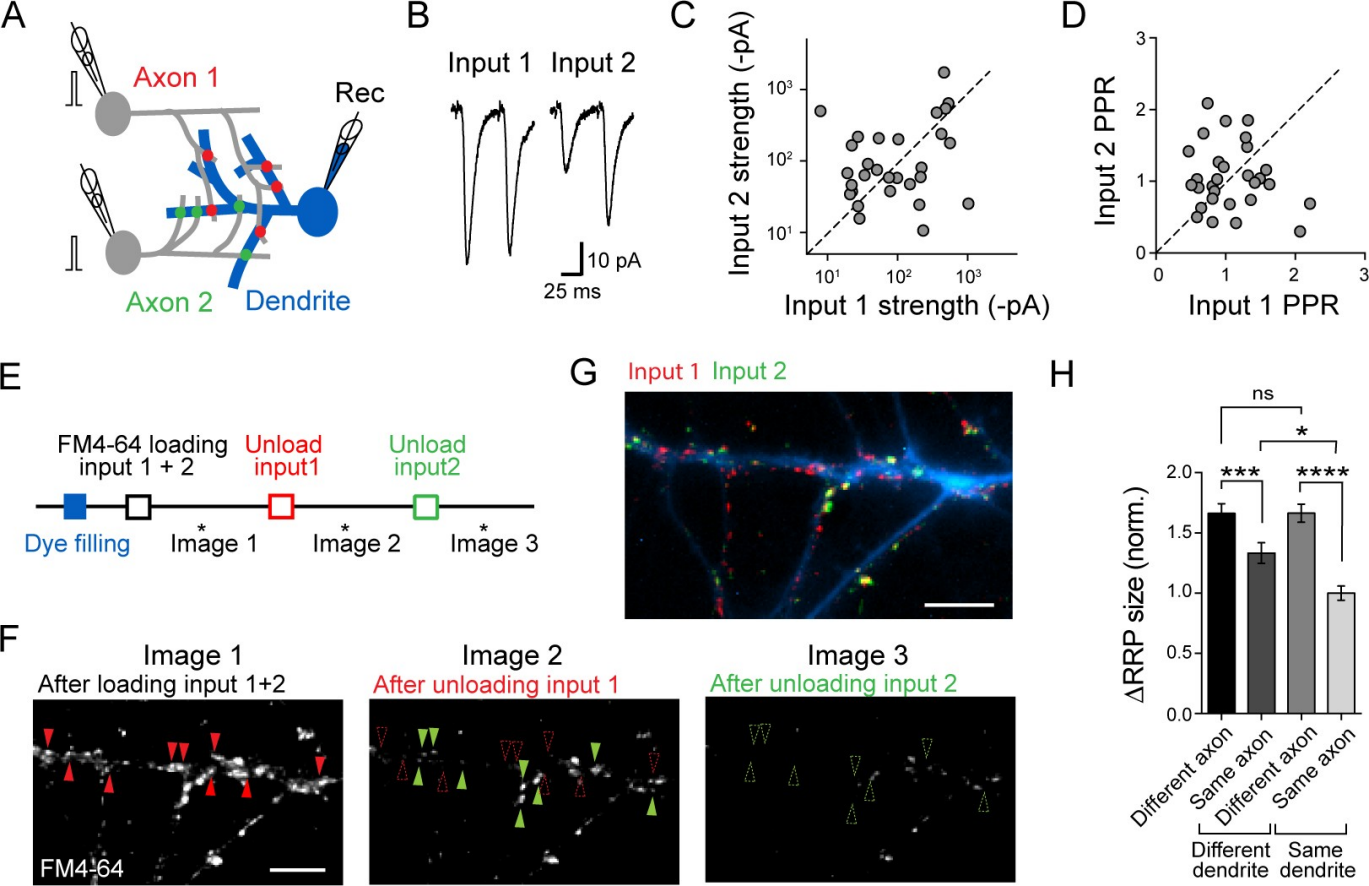
Presynaptic strengths segregate depending on both the dendritic location and the identity of presynaptic inputs. (A) Experimental scheme to compare synaptic inputs from two presynaptic cells on the same postsynaptic neuron. Patch-clamp recordings were used to monitor evoked synaptic responses; FM4-64 dye loading of the presynaptic terminals of each of the two presynaptic cells was used to map the distribution of presynaptic strengths across dendrites of the postsynaptic neuron filled with an Alexa Fluor dye. (B) Example of average EPSC traces to paired stimuli (50-ms interstimulus interval) for each input. (C–D) Scatter plot comparing the EPSC amplitude (C) and PPR (D) of the two inputs. Plots include previously reported data set (*n* = 29 triplets from 12 cultures), which was obtained under exactly the same experimental conditions as the present study (Supplementary Information S7B Fig in [48]). (E) Experimental scheme for estimating RRP size at individual boutons from the two inputs. FM4-64 was loaded by 40 APs at 20 Hz by stimulating presynaptic cells via the patch pipette. Images were acquired before and after unloading the FM4-64 dye with 600 APs at 10 Hz, and the remaining signal was taken as background. (F) Example images of dye-loaded synapses (left), after first (middle) and second (right) unloading stimulations. Scale bar, 5 μm. Red and green arrowheads indicate boutons before (filled) and after (open) the unloading. (G) Image showing the overlay of the fluorescence signals corresponding to each of the two inputs (green and red). The signals for input 1 (red) and input 2 (green) were obtained by subtracting image 2 from image 1 and image 3 from image 2, respectively, as shown in F. Scale bar = 5 μm. (H) Summary graph showing the fluorescence intensity difference for pairs of boutons belonging to the same or different axons and contacting the same or different dendrites (*n* = 150 boutons from 3 triplets, Kruskall–Wallis test followed by Dunn multiple comparison test, *p < 0.05, ***p < 0.001). Underlying data can be found in S1 Data. AP, action potential; EPSC, excitatory postsynaptic current; norm., normalized; ns, not significant; PPR, paired-pulse ratio; Rec, recording pipette; RRP, readily releasable pool.

We first assessed the relative contribution of the presynaptic cell and the postsynaptic dendritic branch in determining the presynaptic efficacy. We took advantage of dissociated cultures in which the unitary connections between identified neurons can be studied at the level of individual synapses [35,60]. In this system, an axon often established multiple synaptic contacts onto several dendritic branches of the postsynaptic neuron, resulting in sparse connectivity (Fig 1A) [35,60], a situation mimicking several in vivo connections [3]. In order to assess how presynaptic cell identity influences synaptic strengths, we compared the properties of synaptic inputs from two independent presynaptic pyramidal neurons making monosynaptic connections onto a same postsynaptic pyramidal neuron and that were themselves not synaptically coupled to each other (Fig 1A). Whole-cell patch-clamp recordings revealed highly variable excitatory postsynaptic current (EPSC) amplitudes that were not correlated between the two convergent inputs (Fig 1B and 1C). To estimate the extent to which the difference in EPSC amplitudes between the two inputs reflected the differences in presynaptic efficacies, we measured the paired-pulse ratio (PPR), a parameter inversely related to the neurotransmitter release probability (*p*_*r*_), where *p*_*r*_ is defined as the likelihood of the occurrence of neurotransmitter release at a presynaptic terminal in response to an action potential [7,61–63]. Similarly to the EPSC amplitudes, PPR was not correlated between the two inputs (Fig 1D; also see S7B Fig in [48]), suggesting that their presynaptic strengths—representing the sum of successful transmitter release events at each synapses of the connection—were distinct despite sharing the common postsynaptic target cell.

In order to test whether this difference was maintained when the two axons contacted the same dendritic branch of the postsynaptic neuron, we filled the postsynaptic neuron with the Alexa Fluor 488 dye and estimated the presynaptic strength of individual boutons apposed to the Alexa Fluor dye-filled dendrite, using the styryl dye FM4-64 (Fig 1E–1G). The two presynaptic neurons were simultaneously stimulated with 40 action potentials (APs) at 20 Hz in the presence of FM4-64 to label the readily releasable pool (RRP) of vesicles, a parameter that has been previously shown to be related to *p*_*r*_ [60,64–67]. In order to assign each labeled bouton to either one of the two presynaptic cells, the two cells were sequentially stimulated with 600 APs at 10 Hz to fully deplete the RRP and induce the loss of FM4-64 fluorescence (unloading) at each bouton (Fig 1E–1G, S1 Video). The difference in FM4-64 fluorescence signal before and after unloading was taken as a measure of the RRP size. Boutons from a same presynaptic neuron displayed more similar RRP size when contacting the same dendritic branch than when contacting different dendritic branches, which was consistent with previous studies showing the dependence of presynaptic efficacy on synapse location on the dendritic tree (Fig 1H) [13,34,35]. However, we also found that boutons had more similar RRP size when coming from the same presynaptic neuron than from different presynaptic neurons, which could reflect inherent differences in the activity of presynaptic neurons; this effect was maintained when the boutons also contacted the same dendritic branch (Fig 1H). These observations suggest that presynaptic strengths are determined by both the identity of the presynaptic neuron and the local dendritic branch properties [9,35,48].

### Postsynaptic strengths primarily depend on the dendritic branch and, locally, on the presynaptic cell identity

**Fig 2:**
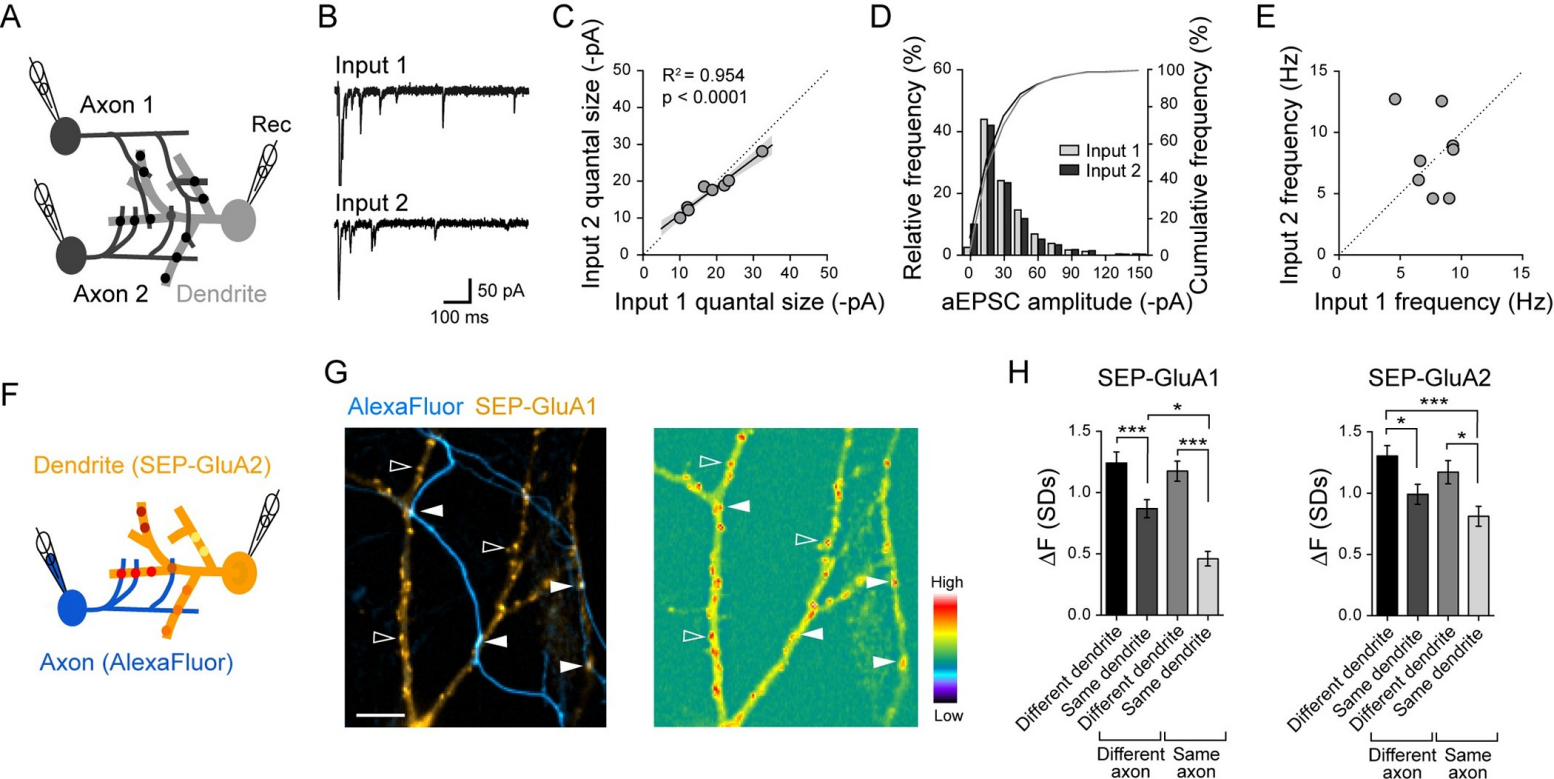
Postsynaptic strengths segregate depending on dendritic branches and locally, on presynaptic inputs. (A) Triple recording scheme with two independent presynaptic neurons forming convergent inputs onto a postsynaptic neuron. (B) Example traces of aEPSCs recorded in Sr^2+^-containing aCSF in response to stimulating each of the two convergent inputs. (C) Comparison of average aEPSC amplitude of two inputs (R^2^ = 0.95, *p* < 0.0001, *n* = 8 triplets). Linear regression and 95% confidence interval (gray) are shown. (D) Histograms from a representative triple recording experiment showing quantal event amplitudes for each of the two convergent inputs (gray and white bars). (E) Comparison of mean aEPSC frequency of two convergent inputs shows no correlation (R^2^ = 0.083, *p* = 0.4885, *n* = 8 triplets). (F) Scheme to probe the distribution of surface AMPA receptors. A postsynaptic neuron expresses SEP-GluA1 or SEP-GluA2 (yellow), and a nearby presynaptic partner is filled with an Alexa Fluor dye (blue). (G) Fluorescence images from a representative experiment. Left, postsynaptic SEP-GluA1–positive dendrites are contacted by an Alexa Fluor dye–labeled axon; some SEP-GluA1 puncta are contacted by the labeled axon (filled arrowheads), and others are presumed to receive synaptic inputs from unlabeled axons (open arrowheads). Right, same view shows punctate SEP-GluA1 signal representing putative postsynapses whose fluorescence intensity is color-coded. Scale bar, 10 μm. (H) Summary graph of the fluorescence intensity difference for pairs of SEP-GluA1 (left) or SEP-GluA2 (right) puncta apposed to different or same axon and belonging to different or same dendrites (SEP-GluA1: *n* = 461 puncta from 8 cells, SEP-GluA2: *n* = 1,066 puncta from 9 cells, one-way ANOVA followed by Tukey multiple comparison test, **p < 0.01, ***p < 0.001). Underlying data can be found in S1 Data. aCSF, artificial cerebrospinal fluid; aEPSC, asynchronous EPSC; EPSC, excitatory postsynaptic current; AMPA, α-amino-3-hydroxy-5-methyl-4-isoxazolepropionic acid; GluA, AMPA receptor subunit; Rec, recording pipette; SEP, superecliptic pHluorin.

We next examined whether postsynaptic strengths of individual synapses were similarly influenced by the dendritic branch location and the presynaptic cell identity. Using the same triplet neuron configuration as above, we first took an electrophysiology approach and compared the amplitude and frequency of quantal responses from two presynaptic cells by evoking asynchronous events in Sr^2+^-containing artificial cerebrospinal fluid (aCSF) [68,69] (Fig 2A and 2B). Surprisingly, the average quantal asynchronous EPSC (aEPSC) amplitude was highly similar between the two inputs, and the corresponding aEPSC amplitude distributions fully overlapped (Fig 2C and 2D). The average aEPSC kinetics were also similar between the two inputs, with no significant differences in the rise or decay times; in addition, we could not detect any systematic effect of the series resistance on aEPSC amplitude or kinetics for each recording (S1 Fig). This suggested that the multiple synapses made by each of the two presynaptic inputs were distributed in an equivalent manner across the dendritic tree and produced, on average, a comparable level of postsynaptic glutamate receptor activation. This raised the possibility that unlike the overall presynaptic strength, the average quantal size was not dependent on the presynaptic neuron identity.

In contrast to the correlated aEPSC amplitudes, the average aEPSC frequency was not related between the two presynaptic inputs (Fig 2E), which was in accord with the observed heterogeneity of EPSCs and PPR between the two inputs in Ca^2+^-containing aCSF (Fig 1C and 1D). The differences in aEPSC frequency between the two inputs could represent variations in the total number of functional synapses and/or the *p*_*r*_ of boutons made by each of the two presynaptic inputs onto the same postsynaptic neuron.

We next used an optical approach to investigate the relative contribution of presynaptic cell identity and dendritic branch location in determining the postsynaptic strength of individual synapses (Fig 2F and 2G). We expressed in postsynaptic neurons either GluA1 or GluA2 AMPA receptor subunits that had been extracellularly tagged with superecliptic pHluorin (SEP) [39,70,71]. Both SEP-GluA1 and SEP-GluA2 showed punctate fluorescence signal along dendrites; the normalized distribution of fluorescence intensity values was similar to the normalized distribution of miniature EPSC (mEPSC) amplitudes (S2 Fig). We therefore considered SEP-GluA1 or SEP-GluA2 puncta as postsynaptic markers and their fluorescence intensity as a proxy for postsynaptic strength or quantal size. Pairwise comparisons of SEP-GluA1 or SEP-GluA2 puncta intensity revealed a smaller fluorescence intensity difference for puncta sharing the same dendritic branch compared to those on separate dendritic branches (Fig 2H). This finding is consistent with the idea that postsynaptic strengths are set locally according to the dendritic branch [22,36,38–40,46]. Interestingly, the SEP-GluA1 or SEP-GluA2 fluorescence was also more similar for puncta contacted by the same presynaptic cell filled with the Alexa Fluor 594 dye, but this effect was observed only when they also shared the same dendritic branch (Fig 2H). This suggested that postsynaptic strengths depend primarily on the dendritic location and less so on the presynaptic cell identity. This was in agreement with the analysis of aEPSCs in which the amplitude histograms and the average amplitudes did not show a difference between the two presynaptic inputs (Fig 2C and 2D).

### Induction of plasticity normalizes presynaptic strengths in the stimulated axon

**Fig 3:**
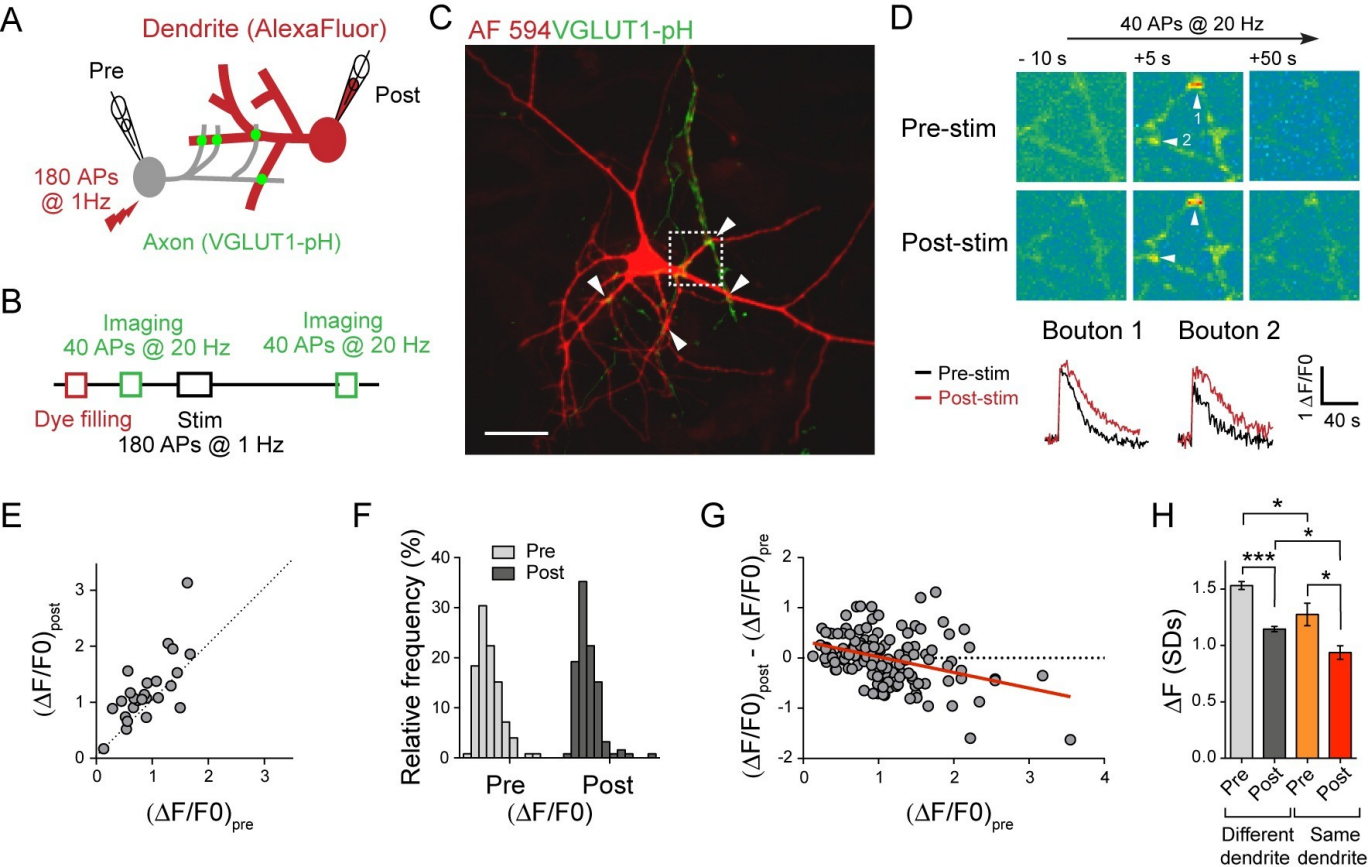
Activity normalizes presynaptic strengths in the stimulated axon. (A) Scheme for imaging synaptic vesicle dynamics at individual boutons. A presynaptic neuron expressing VGLUT1-pHluorin (green) and a postsynaptic partner filled with an Alexa Fluor 594 dye (red) were patch clamped. (B) Experimental scheme for monitoring activity-dependent changes in the RRP size of individual boutons contacting the postsynaptic neuron as shown in (A). (C) Fluorescence image showing Alexa Fluor dye–filled dendrites (red) contacted by VGLUT1-pHluorin–positive puncta (green). Putative synaptic contacts are indicated by arrowheads. Scale bar, 30 μm. (D) An example of eliciting presynaptic plasticity in a single connection. Images of VGLUT1-pHluorin fluorescence change (top) and fluorescence intensity traces (bottom) at indicated boutons (arrowheads) triggered by a 40-AP, 20-Hz stimulation are shown before and 20 min after the conditioning stimulation to elicit plasticity. (E) Plot comparing the RRP size before and after the conditioning stimulation for individual boutons from the example shown in (C,D) (*n* = 25). (F) Comparison of the distribution of RRP size before and after the conditioning stimulation for all boutons analyzed (*n* = 134 boutons from 7 cells). (G) Summary plot comparing the change in RRP induced by the conditioning stimulation versus the initial RRP size (R^2^ = 0.13, *p* < 0.0001, *n* = 134 boutons from 7 cells). The red line represents the linear regression. (H) Summary of RRP size difference analysis for pairs of boutons apposed to different dendritic branches or the same dendritic branch, before and after the conditioning stimulation, expressed as mean ± SEM (one-way ANOVA followed by Tukey multiple comparison test, *p < 0.05, ***p < 0.001). Underlying data can be found in S1 Data. AF, Alexa Fluor; AP, action potential; RRP, readily releasable pool; stim, stimulation; VGLUT1, vesicular glutamate transporter 1

Our experiments thus far examined the relative contribution of the presynaptic cell and the dendritic branch in controlling pre- and postsynaptic strengths under basal conditions. We next investigated how activity of the presynaptic neuron affects the relative presynaptic strengths of the boutons within the stimulated axon and with respect to their dendritic location. We previously found that eliciting APs at 1 Hz for 3 min in 1 presynaptic cell with the postsynaptic cell under current-clamp (conditioning stimulation) induced long-term changes in presynaptic strengths of both stimulated and nonstimulated axons that shared the same target postsynaptic neuron [48]. Notably, the same stimulation could elicit either potentiation or depression in a manner that promoted the overall presynaptic strength heterogeneity through a mechanism involving astrocytes [48]. Here, we performed a meta-analysis of the presynaptic strengths data set reported in our previous study (S2 Fig in [48]). Briefly, synaptic vesicle dynamics at individual boutons was directly visualized by expressing vesicular glutamate transporter 1 (VGLUT1) tagged with a pH-sensitive variant of green fluorescent protein (GFP) in its luminal domain (VGLUT1-pHluorin) [72]. Dual patch-clamp recordings were made between a presynaptic neuron expressing VGLUT1-pHluorin and a nearby nontransfected postsynaptic neuron filled with an Alexa Fluor dye to visualize the dendritic branches (Fig 3A–3C). To determine how the induction of plasticity by the conditioning stimulation affected presynaptic strengths of individual boutons contacting the postsynaptic cell, we assessed the RRP size by stimulating the presynaptic neuron with 40 APs at 20 Hz, before and after the conditioning stimulation (Fig 3B). The resulting increase in the VGLUT1-pHluorin fluorescence relative to the baseline (ΔF/F_0_) caused by exocytosis and recycling of the RRP vesicles represented the RRP size [35,72,73] (Fig 3D, S2 Video). In agreement with the FM4-64 dye experiments (Fig 1), RRP sizes of boutons from the same axon on the target neuron were highly heterogeneous under basal conditions (Fig 3E–3H), but they were more similar if the boutons were apposed to the same dendritic branch (Fig 3H). Applying the 1-Hz stimulation for 3 min to induce plasticity resulted in heterogeneous changes of the RRP size within the same connection (Fig 3E–3H). Notably, we observed a significant trend for synapses that had a lower RRP before the induction of plasticity to increase their RRP size and vice versa (Fig 3F and 3G), which reduced the width of the RRP distribution (Fig 3F: coefficient of variation [CV] before the stimulation: 56.4 ± 4.5; CV after the stimulation: 44.5 ± 4.4, Wilcoxon matched pairs signed-rank test, *p* < 0.05) and effectively resulted in the normalization of RRP of synapses formed between the pre- and the postsynaptic neuron. Interestingly, the spatial distribution of RRP sizes on the dendrite was maintained after the stimulation (Fig 3H), suggesting that the mechanism of normalization might act independently of the local dendritic retrograde regulation of release probability that favors boutons sharing the dendritic branch to have more similar RRP [35].

### Induction of presynaptic plasticity accompanies a rapid and uniform downscaling of postsynaptic strengths in primary hippocampal cultures

**Fig 4:**
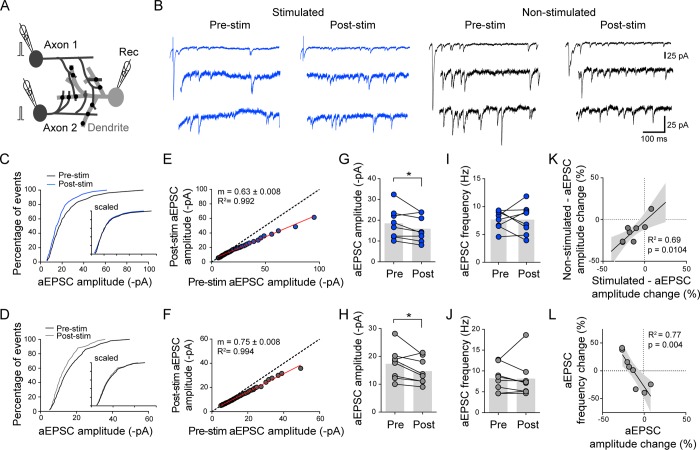
Activity-dependent downscaling of postsynaptic strengths compensates for presynaptic potentiation. (A) Experimental scheme to compare the effect of the conditioning stimulation on quantal properties of convergent inputs in Sr^2+^-containing aCSF. The conditioning stimulation (1 Hz, 3 min) is given to one of the two presynaptic cells in Ca^2+^-containing aCSF. (B) Example traces of aEPSCs recorded in Sr^2+^-containing aCSF for stimulated (blue) and nonstimulated (black) input, before and >15 min after the conditioning stimulation. The trace on the top includes the first synchronous peak (low scale); the two bottom traces show asynchronous quantal events (high scale). (C,D) Cumulative distributions of stimulated (C) and nonstimulated (D) aEPSC amplitude before and >15 min after the conditioning stimulation. Insets show the scaled cumulative distributions. (E,F) Summary plots showing the comparison of rank-ordered aEPSC amplitudes after versus before the stimulation at stimulated (E: R^2^ = 0.99, *p* < 0.0001) and nonstimulated (F: R^2^ = 0.99, *p* < 0.0001) inputs (*n* = 8 experiments). Linear regression is shown in red where m indicates the slope. (G–J) Summary of mean aEPSC amplitude (G,H) and frequency (I,J) before and after the stimulation at stimulated (G,I) and nonstimulated (H,J) inputs (*n* = 8 triplets). Data were evaluated using the paired, two-tailed Student *t* test. *p < 0.05. (K) Comparison of the percent change between stimulated and nonstimulated connections in mean aEPSC amplitude (R^2^ = 0.69, *p* = 0.0104), with linear regression (black line) and 95% confidence interval (gray). (L) Comparison of the percent change between aEPSC amplitude and aEPSC frequency for the stimulated connection (R^2^ = 0.77, *p* = 0.004), with linear regression (black line) and 95% confidence interval (gray). Underlying data can be found in S1 Data. aCSF, artificial cerebrospinal fluid; aEPSC, asynchronous EPSC; EPSC, excitatory postsynaptic current; Rec, recording pipette; stim, stimulation.

We next sought to determine how the conditioning stimulation of a single presynaptic neuron that induces presynaptic plasticity affects postsynaptic strengths of the target neuron. To this end, we again performed patch-clamp recordings in the triplet neuron configuration and assessed the effects of stimulating 1 of the 2 presynaptic cells at 1 Hz for 3 min on aEPSC amplitude and frequency of both the stimulated and nonstimulated inputs (Fig 4A). aEPSCs were monitored in Sr^2+^-containing aCSF before and more than 15 min after the conditioning stimulation, which was applied in Ca^2+^-containing aCSF (Fig 4B). Surprisingly, the conditioning stimulation moderately but consistently decreased the mean aEPSC amplitude at both stimulated and nonstimulated inputs (-13.3 ± 4.4% and -13.9 ± 4.7%, respectively: Fig 4B, 4G, 4H and 4K). Furthermore, the decrease was multiplicative at both inputs as illustrated by the uniform scaling of the aEPSC amplitude cumulative histogram (Fig 4C and 4D; also see 4E and 4F). In contrast, aEPSC frequency changes were variable (Fig 4I and 4J) and unrelated between the two inputs for individual experiments (S3 Fig), in agreement with our previous study, in which the changes in PPR associated with plasticity induction were not strongly correlated between convergent inputs [48]. Notably, the depression of aEPSC amplitudes induced by the conditioning stimulation was blocked by perfusing 10 mM 1,2-bis(o-aminophenoxy)ethane-N,N,N′,N′-tetraacetic acid (BAPTA) into the postsynaptic neuron via the patch pipette to chelate intracellular calcium (S4 Fig). Therefore, the observed depression of postsynaptic strengths was not input specific, although it likely required postsynaptic calcium signaling.

**Fig 5:**
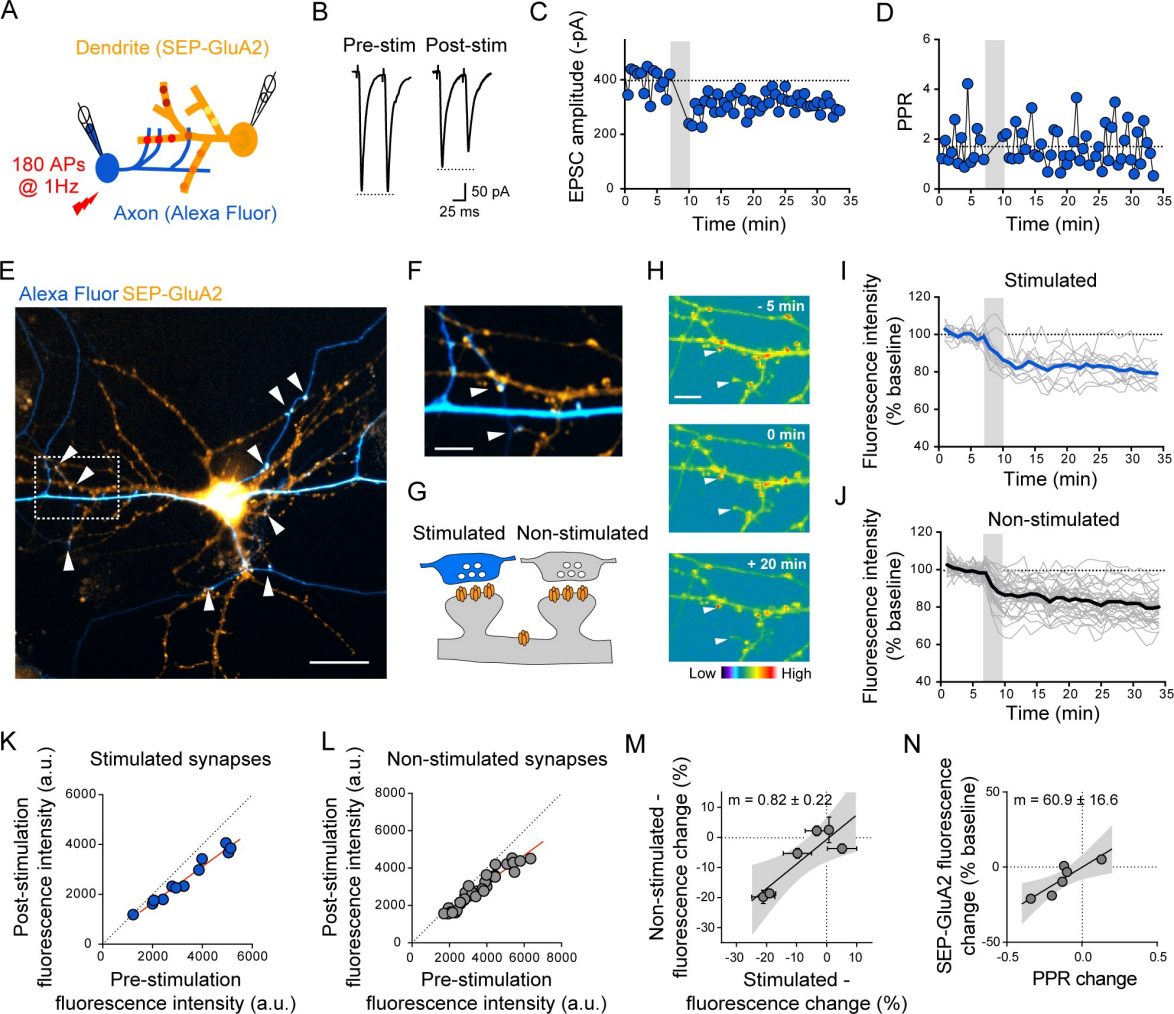
Uniform downscaling of postsynaptic AMPA receptors across the dendritic tree in primary hippocampal cultures. (A) Scheme to visualize individual postsynaptic strengths of stimulated and nonstimulated synapses. (B) Representative EPSC trace evoked by a pair of brief depolarizations (+100 mV, 50-ms interval) of the presynaptic neuron. (C–D) Time course of EPSC amplitude (C) and PPR (D) from an example recording. (E) Fluorescence image of a SEP-GluA2–expressing postsynaptic neuron (yellow) receiving putative synaptic contacts from an axon of a neuron filled with an Alexa Fluor dye (blue). Scale bar, 40 μm. (F) Details of the boxed area in (E). Arrowheads indicate putative synaptic contacts between the stimulated presynaptic cell and the postsynaptic cell. Scale bar, 10 μm. (G) Illustration of the basis for discriminating between stimulated and nonstimulated synapses. SEP-GluA2 puncta apposed to the labeled axon are considered as stimulated synapses, while the others are considered as nonstimulated. (H) Time-lapse images of color-coded fluorescence intensity showing the decrease of SEP-GluA2 signal after the conditioning stimulation for the area corresponding to (F). Individual SEP-GluA2 puncta associated to the labeled axon shown in (F) are indicated (arrowheads). Scale bar, 10 μm. (I,J) Time course of SEP-GluA2 fluorescence intensity at putative stimulated synapses (I: paired, two-tailed Student *t* test, ****p < 0.0001, *n* = 12 synapses) and nonstimulated synapses (J: paired, two-tailed Student *t* test, ****p < 0.0001, *n* = 34 synapses) for the same postsynaptic neuron. (K,L) Plots of the SEP-GluA2 fluorescence intensity before versus after the stimulation at stimulated synapses and nonstimulated synapses for the same example in (E–J) (K: R^2^ = 1.00; L: R^2^ = 0.92, *p* < 0.0001). (M) Comparison of the extent change in fluorescence intensity of SEP-GluA2 at stimulated versus nonstimulated synapses (R^2^ = 0.77, *p* = 0.0209, *n* = 6 cells), with linear regression shown. Data are expressed as mean ± SEM. m indicates the slope. (N) Comparison of the extent change in fluorescence intensity of SEP-GluA2 versus PPR change at stimulated synapses (R^2^ = 0.77, *p* = 0.0209, *n* = 6 cells), with linear regression shown. m indicates the slope. Underlying data can be found in S1 Data. AMPA, α-amino-3-hydroxy-5-methyl-4-isoxazolepropionic acid; AP, action potential; a.u., arbitrary unit; EPSC, excitatory postsynaptic current; GluA, AMPA receptor subunit; PPR, paired-pulse ratio; SEP, superecliptic pHluorin; stim, stimulation.

We then tested whether the apparent multiplicative downscaling of postsynaptic strengths induced by the conditioning stimulation could be detected optically at individual synapses by expressing SEP-GluA2 in the postsynaptic neuron. Dual patch-clamp recordings of the SEP-GluA2–expressing postsynaptic neuron with a neighboring presynaptic partner filled with the Alexa Fluor 594 dye enabled us to simultaneously monitor the EPSCs while imaging individual SEP-GluA2 puncta. SEP-GluA2 puncta apposed to the dye-labeled axon were considered as stimulated synapses, while the others not associated to the labeled axon were considered as nonstimulated synapses (Fig 5A). Upon applying the conditioning stimulation, in 3 out of 6 paired recordings, SEP-GluA2 signal intensity decreased by comparable extents at both stimulated and nonstimulated synapses (-16.6 ± 3.5% and -14.6 ± 4.6%, respectively; Fig 5E–5M; S5A, S5B and S5D Fig), while in one recording, the SEP-GluA2 intensity remained stable over time (S5C Fig).

Collectively, these observations at the level of individual synapses indicated that postsynaptic depression, when it occurred, was not restricted to stimulated synapses. Moreover, the 1-Hz, 3-min stimulation did not promote potentiation of quantal amplitudes even though an increase in RRP could be observed in stimulated axons. In contrast to the quantal amplitude, the EPSC amplitude representing the global strength of the stimulated input showed long-lasting potentiation, depression, or no change (Fig 5B–5D and S5 Fig), displaying a variable outcome as observed for the presynaptic changes [48].

### The postsynaptic downscaling counterbalances presynaptic potentiation

The combined live imaging of SEP-GluA2 and patch-clamp recordings at identified connections before and after the conditioning stimulation provided us with the opportunity to probe the relationship between the overall plasticity outcome (EPSC amplitude change) and presumed presynaptic (PPR) and postsynaptic (SEP-GluA2) changes. Surprisingly, for individual connections, the average decrease in SEP-GluA2 signal intensity at stimulated synapses was mirrored by a decrease in PPR (Fig 5N). This suggested that postsynaptic depression might counterbalance to some degree the presynaptic potentiation elicited at the stimulated connection, which would result in no net change of EPSC amplitude (S5D Fig). Furthermore, depression of EPSC amplitude could be accompanied by a decrease in SEP-GluA2 signal intensity with no change or an increase in PPR (S5A and S5B Fig). In contrast, potentiation of EPSC amplitudes was observed with a decrease in PPR but without a change in SEP-GluA2 intensity in one experiment (S5C Fig). Altogether, these observations suggested that the global synaptic plasticity outcome could be predicted by a relative balance in the changes in PPR or SEP-GluA2 signal intensity.

We next asked whether the negative correlation between pre- and postsynaptic changes associated with plasticity induction was also observed for the changes in aEPSC frequency and amplitude recorded in Sr^2+^ (Fig 4). Consistent with the analysis of the changes in PPR and SEP-GluA2 intensity, we found that a large increase in aEPSC frequency was accompanied by a large decrease in aEPSC amplitude (Fig 4L). This suggested that a global postsynaptic depression could counteract presynaptic potentiation and vice versa and contribute to the variability in the direction and the extent of EPSC amplitude changes.

### Characterizing features of unitary synaptic connections between identified CA3 pyramidal cells in organotypic slices

**Fig 6:**
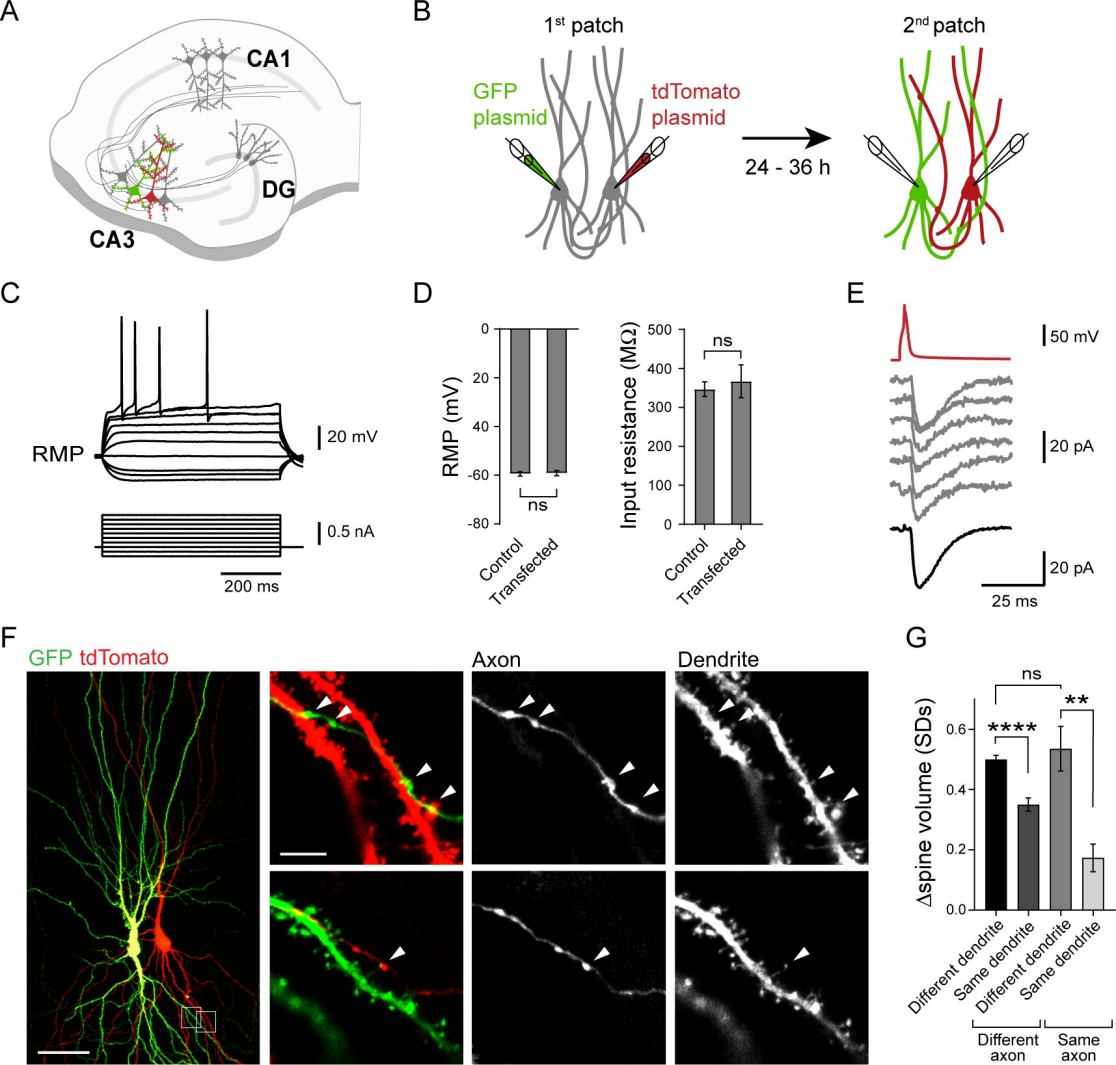
Spine head volume of CA3 cells segregate depending on dendritic branches and, locally, on presynaptic CA3 inputs. (A) Scheme illustrating a single pair of CA3 pyramidal cells from an organotypic slice that have been transfected to express GFP and tdTomato. (B) Experimental strategy to express different fluorescent proteins in a pair of CA3 cells. Whole-cell recordings are used to ensure the functional connectivity between the two cells and to infuse plasmids encoding GFP and tdTomato, allowing for visualization and discrimination of their axons and dendrites 24–36 h later. (C) Traces showing current-voltage relationship from a transfected CA3 pyramidal cell (100 pA current injection steps for 600 ms, from -200 pA to +700 pA). (D) Summary of RMP and Ri of transfected cells and control cells in same recording conditions (transfected: *n* = 10 cells, control *n* = 20 cells, Mann–Whitney test). (E) Example traces recorded from a connected CA3 cell pair showing synaptic currents (gray traces) evoked in response to an AP (red trace) triggered in the presynaptic cell 24–36 h after the transfection (second patch). The thick black trace at the bottom represents the average EPSC trace. (F) Confocal images acquired with a scanning laser microscope showing morphological interactions between CA3 neurons from a slice fixed 24–36 h after transfection. Left panel shows low magnification image of the 2D projection of a confocal stack. Right panels show high magnification views of the morphological contacts (arrowheads) between axonal boutons and dendritic spines from the CA3 neurons expressing GFP or tdTomato. Scale bar: Left panel, 50 μm; right panels, 5 μm. (G) Summary graph showing the spine head volume difference for pairs of spines apposed to different or same axon and belonging to different or same dendritic segments (*n* = 318 spines from 5 pairs, Kruskall–Wallis test followed by Dunn multiple comparison test, *p < 0.05, ****p < 0.0001). Underlying data can be found in S1 Data. AP, action potential; CA3, Cornu Ammonis 3; DG, dentate gyrus; EPSC, excitatory postsynaptic current; GFP, green fluorescent protein; ns, not significant; Ri, input resistance; RMP, resting membrane potential.

We next sought to examine whether our observations in sparsely connected dissociated hippocampal neurons displaying random connectivity (but see [74]) could be reproduced in a more physiological model. To this end, we turned to the hippocampal Cornu Ammonis 3 (CA3) recurrent network, which shows sparse and uniform connectivity and is thought to enable efficient storage and retrieval of associative memories [75–77]. We set up an experimental approach to monitor putative individual synaptic contacts in functionally connected CA3 pyramidal cell pairs in days in vitro (DIV) 18–25 hippocampal organotypic slices. We performed whole-cell recordings to confirm the functional connectivity between two cells, and during the recordings, plasmids encoding GFP and tdTomato were infused for subsequent visualization and discrimination of their axons and dendrites (Fig 6A and 6B) [78]. At the time of transfection, monosynaptic AMPA-receptor–mediated currents could be elicited in 27 out of 48 CA3–CA3 pairs (56%) recorded, in agreement with previous studies [79,80]: 18 pairs (67%) were unidirectionally connected, and 9 (33%) were connected in both directions. Unitary EPSCs had a mean latency of 3.3 ± 0.4 ms, an average maximal EPSC amplitude (excluding events with failures) of 18.5 ± 1.9 pA, a rise time of 1.7 ± 0.1 ms, and a decay time of 10.3 ± 0.7 ms (S6A Fig), which were similar to previous reports for CA3–CA3 recurrent connections in organotypic and acute slices [77,79,81]. After the recording, the patch pipettes were slowly retracted to facilitate membrane resealing. Slices were then returned to the incubator for another 24–36 h to allow for the expression of GFP and tdTomato. Transfected cells survived and displayed typical CA3 morphology and excitability (Fig 6C and 6D), with comparable resting membrane potential (RMP) and input resistance (Ri) to nontransfected control cells (RMP: transfected = -59.4 ± 0.9 mV; control = -59.2 ± 1.0 mV; Ri: transfected = 367 ± 42 MΩ; control = 347 ± 19 MΩ; Fig 6D). All rerecorded pairs (*n* = 10) remained functionally connected as monitored 24–36 h earlier (Fig 6E, S6B Fig). The average EPSC peak amplitude of unitary connections, which excluded events with failures, remained comparable to the first patch-clamp recording, suggesting that the average number of synaptic contacts per connection remained stable; EPSC latency, rise time, and decay time also remained stable (S6B Fig). There was, however, a notable increase in the reliability with which evoked EPSCs could be elicited, which suggested an increase in release probability over the 24–36 h of incubation. In some cell pairs, we observed an increase in the maximal EPSC amplitude (excluding failures), which could involve the conversion of silent synapses [82] and/or new synapse formation. Overall, transfection and expression of fluorescent probes via whole-cell recording did not noticeably disrupt the health of neurons and therefore allowed for a closer examination of the properties of synaptic connection between identified CA3 cell pairs.

### Spine head volume of CA3 pyramidal cell primarily depends on dendritic location and, locally, on the presynaptic cell identity

Slices in which a single pair of connected CA3 cells was labeled with GFP and tdTomato were fixed for subsequent analysis of individual spines across the dendritic tree using confocal laser scanning microscopy. Functionally connected CA3 pairs showed between 1 and 14 putative synaptic contacts that were identified by the apposition of labeled axons with labeled spines on basal and/or apical dendrites (Fig 6F). Similar to dissociated cultured neurons, a single axon could contact spines across different dendritic branches or several spines within the same dendritic branch (Fig 6F).

For each connected cell pair, the spatial distribution pattern of postsynaptic strengths was examined using the spine head volume as a proxy for the abundance of functional AMPA receptors [8]; pairwise comparisons of spine head volumes were made according to their dendritic location and association to the labeled presynaptic cell axon. Spines showed more similar head volumes when they belonged to the same dendritic branch (<20 μm) as compared to different dendritic branches (Fig 6G). Interestingly, spine head volumes were also more similar when contacted by the same presynaptic cell, but only if they were on the same dendritic branch (Fig 6G). These findings in organotypic slices are consistent with the analysis of SEP-GluA fluorescence intensity in dissociated cultures and further support the view that a presynaptic cell can influence the specification of postsynaptic strengths only locally, at the level of a single dendritic branch.

### LTD at unitary CA3 recurrent connections accompanies decreases in PPR and spontaneous EPSC amplitudes

**Fig 7:**
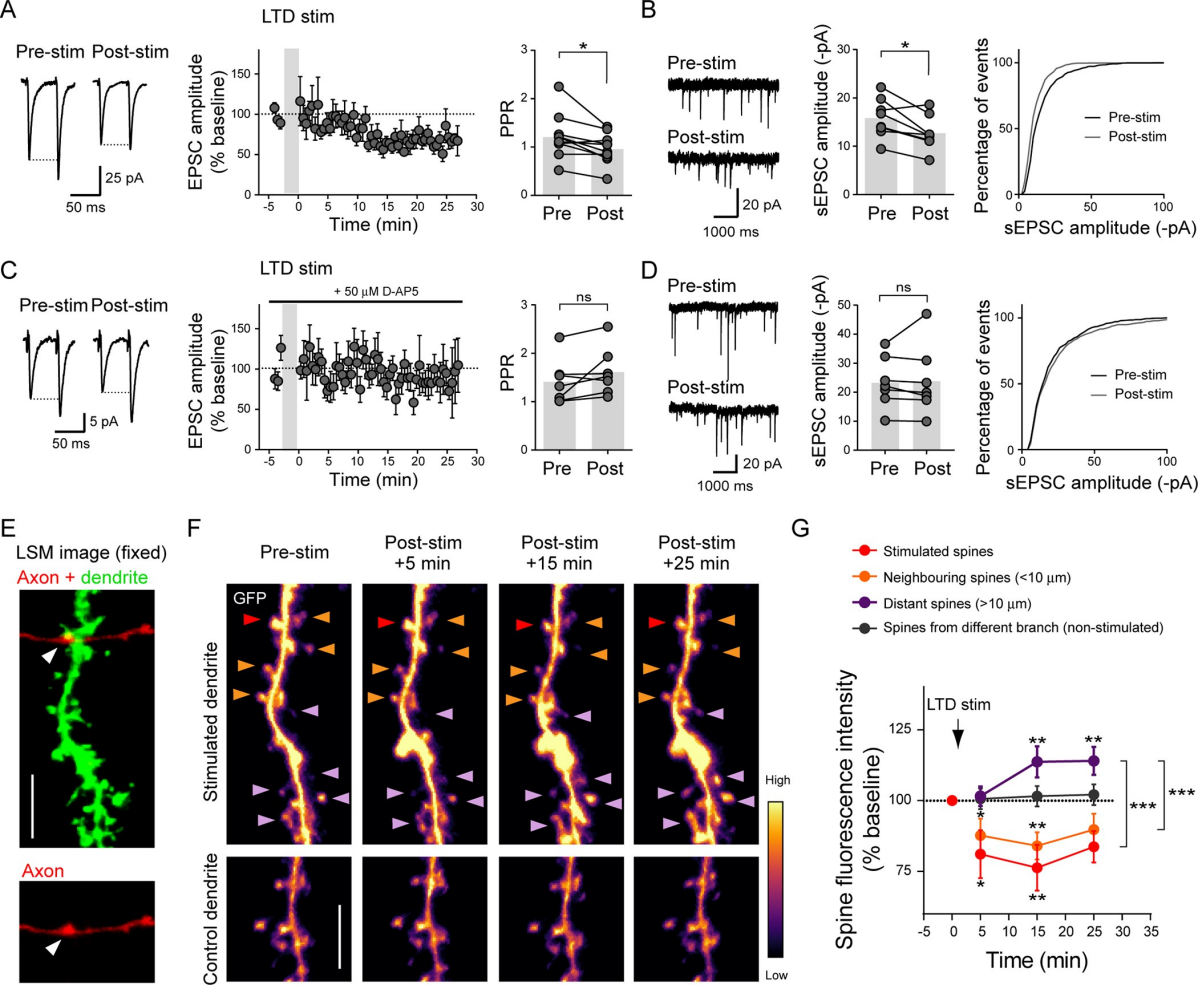
LTD induction at unitary CA3 recurrent connections triggers functional and structural plasticity at both stimulated and nonstimulated synapses. (A,C) Left: representative traces showing synaptic currents in the postsynaptic CA3 neuron evoked by a pair of APs triggered in the presynaptic CA3 neuron (2–3 nA, 50-ms interval), before and 20 min after LTD induction in absence (A) or presence (C) of 50 μM D-AP5. Middle: summary of the time course of EPSC amplitude (*n* = 10 cell pairs) for untreated (A) and D-AP5 (C). The gray shaded box represents the LTD induction. Right: graph plots showing PPR values before and after LTD induction in absence (A) or presence (C) of D-AP5 (untreated: *n* = 10 cell pairs; D-AP5: *n* = 7 cell pairs; paired two-tailed Student *t* test, *p < 0.05). (B,D) Left: example traces of sEPSCs recorded before (Pre-stim) and after (Post-stim) LTD induction in absence (B) or presence (D) of 50 μM D-AP5. Middle: graph plots showing sEPSC amplitude before and after LTD induction in absence (B) or presence (D) of D-AP5 (untreated: *n* = 9 cell pairs, D-AP5: *n* = 7 cell pairs, paired two-tailed Student *t* test, *p < 0.05). Right: cumulative distributions of sEPSC amplitudes before and after LTD induction in absence (B) or presence (D) of D-AP5. (E) 2D projection of optical sections acquired with an LSM from a slice fixed after the live-imaging session. The image shows a dendritic segment (green) with a spine apposed to a presynaptic terminal (arrowhead) of the labeled presynaptic CA3 cell axon (red). Scale bar, 5 μm. (F) 2D projections of time-lapse spinning disk confocal images acquired before (Pre-stim) and 5 min, 15 min, or 25 min after LTD induction and whose fluorescence intensity is color-coded. Images on the top show the same dendritic segment as in (E). The stimulated spine, the neighboring spines, and the distal spines are indicated with red, orange, and purple arrowheads, respectively. The image on the bottom shows a segment of dendrite with no spines contacted by the axon (control dendrite). Scale bar, 5 μm. (G) Time course of spine fluorescence integrated intensity normalized to the baseline for corresponding spines (stimulated, *n* = 9 spines from 4 slices; neighboring, 26 spines from 4 slices; distant, *n* = 28 spines from 4 slices; nonstimulated dendrite, *n* = 27 spines from 4 slices; two-way ANOVA test followed by Tukey and Dunett multiple comparison tests, *p < 0.05, **p < 0.01, ***p < 0.001). Underlying data can be found in S1 Data. AP, action potential; CA3, Cornu Ammonis 3; D-AP5, D-2-amino-5-phosphonovalerate; EPSC, excitatory postsynaptic current; LSM, laser scanning microscope; LTD, long-term depression; ns, not significant; PPR, paired-pulse ratio; sEPSC, spontaneous EPSC; stim, stimulation.

We next investigated how low-frequency stimulation protocols to induce either N-methyl-D-aspartate (NMDA) receptor–dependent long-term depression (LTD) or LTP [83–85] could alter the pre- and the postsynaptic strengths of unitary CA3–CA3 connections. LTD was elicited using a previously reported protocol by stimulating the presynaptic cell at 3 Hz for 3 min with the postsynaptic cell under current clamp [83]. This produced a robust 30% depression of the evoked EPSC amplitude, which lasted for >25 min (Fig 7A). Surprisingly, this depression was accompanied by a consistent decrease in PPR, which suggested an increase in *p*_*r*_. The magnitude of the PPR change was inversely related to the initial PPR and the EPSC amplitude change (Fig 7A and S7 Fig). In addition, the amplitude of spontaneous EPSCs, which likely included responses not necessarily from the stimulated input but from other active inputs, was decreased by 20% (Fig 7B). This suggested the possibility that depression could spread to nonstimulated synapses. Importantly, blocking NMDA receptors with D-2-amino-5-phosphonovalerate (D-AP5) inhibited the depression of both evoked and spontaneous EPSC amplitudes as well as the decrease in PPR (Fig 7C and 7D and S7 Fig).

To elicit LTP, we stimulated the presynaptic cell at 2 Hz for 100 s while maintaining the postsynaptic neuron at 0 mV in voltage-clamp mode [85]. This protocol induced a 200% increase in EPSC amplitude that lasted for >25 min, but potentiation was not accompanied by any consistent change in PPR nor a change in spontaneous EPSC (sEPSC) amplitudes (S7C–S7E Fig). Thus, in contrast to LTD, the synaptic change elicited by LTP induction was primarily expressed postsynaptically and confined to the stimulated spines.

### LTD at unitary CA3-CA3 connections is associated with shrinkage of stimulated and neighboring spines but enlargement of more distant spines

Finally, to investigate whether LTD induction at identified synapses affected neighboring spines, we carried out fluorescence live-imaging experiments on connected CA3 cell pairs labeled with GFP and tdTomato. Using spinning disk microscopy in combination with electrophysiology, we monitored changes in the spine head volume triggered by LTD induction by following the integrated spine fluorescence signal that was normalized relative to the shaft. The 3-Hz, 3-min stimulation of the presynaptic neuron rapidly decreased (by 15%) the fluorescence intensity of spines contacting the stimulated axon (Fig 7E and 7F). The fluorescence intensity of neighboring spines (<10 μm) also decreased to some extent (by 10%) with a similar time course as stimulated spines (Fig 7E and 7F). However, surprisingly, the fluorescence intensity of more distant spines on the same dendritic branch (>10 μm) tended to increase by 15%; this effect was apparent by 15 min to 30 min after LTD induction (Fig 7E and 7F). The average fluorescence intensity of spines from different dendritic branches remained unchanged, suggesting that heterosynaptic plasticity of spine head volume was spatially confined within the stimulated dendritic branch. Together, these results suggest the existence of local signaling that coordinates the heterosynaptic structural remodeling, in which the LTD signal spreads over short distances and then reverts into a potentiating signal at distal spines along the activated dendritic branch.

## Discussion

**Fig 8:**
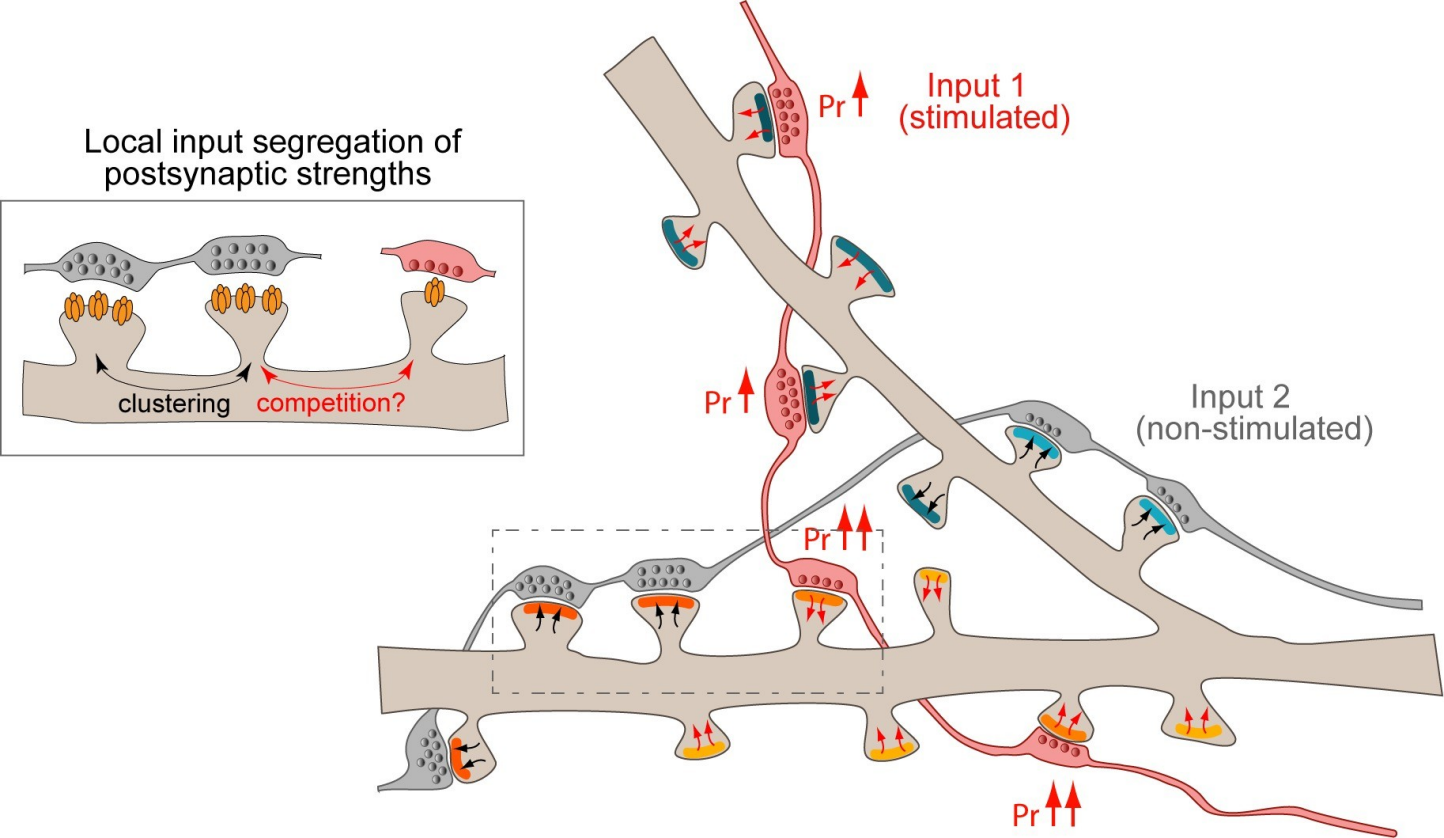
Model for the activity-dependent distribution of pre- and postsynaptic strengths across incoming axons and dendrites. Two axons (input 1 and input 2 in red and gray, respectively) contact several spines from two dendritic branches of the same target neuron. Input specificity of synaptic strengths is best represented at the level of the dendritic branch, where both pre- and postsynaptic strengths segregate depending on the specific presynaptic cells, suggesting that synapses interact locally to compete for or share local resources. In the schematic, different postsynaptic strengths are represented using different shades of blue or orange, and different presynaptic strengths are represented with a different number of presynaptic vesicles. The input-specific stimulation of a single presynaptic cell (red axon) results in the normalization of presynaptic strengths along the axon. The magnitude of presynaptic potentiation (or increased *p*_*r*_, indicated by upward red arrows) depends on the initial presynaptic strength. On the postsynaptic side, the long-lasting presynaptic potentiation accompanies a postsynaptic depression (inward red arrows) that spreads to nonstimulated neighboring spines and that may revert into potentiation at more distant spines (outward black arrows). *p*_*r*_, neurotransmitter release probability.

Our study clarifies the respective roles of pre- and postsynaptic neurons in setting synaptic strengths in the following two aspects. First, the input specificity of incoming information is best reflected at the level of the single dendritic branch, where the presynaptic strengths and, to some degree, the postsynaptic strengths depend on the identity of presynaptic neurons (Fig 8). This further underscores the role of the dendritic branch as a fundamental computational unit that can discriminate and integrate information from distinct presynaptic neurons [37,40,41,86]. Second, our imaging data in both dissociated cultures and organotypic slices reveal that postsynaptic strengths primarily depend on the local dendrite rather than the identity of the presynaptic cell itself. This provides an explanation for our patch-clamp recordings of aEPSCs in dissociated cultures, in which convergent connections, whose synapses are broadly distributed across the dendritic tree of the target postsynaptic neuron, show similar average quantal size (e.g., Figs 3C and 5E). In other words, different inputs making multiple synapses in similar numbers onto multiple dendrites are expected to contribute similarly to somatic depolarization and postsynaptic cell firing in response to single APs. These observations strengthen the idea that differences in synaptic strengths across connections critically depend on the number and also the dendritic location of the synaptic contacts made by each presynaptic input [52,87,88]. In turn, the pattern of spatial distribution of the synaptic strengths of each input will ultimately affect how individual inputs are integrated in the postsynaptic neuron—whether distributed or clustered—to produce the output signal [40,45,88,89].

That the similarity of postsynaptic strengths are spatially confined to the level of dendritic branch but not cell-wide suggests the existence of local interactions between the postsynaptic compartments of different presynaptic inputs, e.g., via signaling along the dendritic shaft [21,22] (Fig 8). Our plasticity experiments in both dissociated cultures and organotypic slices are consistent also with the involvement of heterosynaptic (or intersynaptic) dendritic signaling, in which LTD elicited at individual connections spreads to neighboring spines in a Ca^2+^- and NMDA-receptor–dependent manner. Notably, in slice culture experiments, while the sEPSC amplitude decrease suggests a widespread synaptic depression, the decrease in spine head volume is spatially confined, and instead, potentiation of spine head volume is observed at more distal nonstimulated spines. Such differences between quantal amplitude changes and spine structural plasticity could be attributed to plasticity-driven changes in the composition of synaptic AMPA receptors or the components of the synaptic scaffold that are not necessarily coupled to spine structural changes [90]. Alternatively, sEPSCs could be biased towards inputs activated during the LTD protocol and also affected by the internalization of extrasynaptic AMPA receptors following LTD induction [69]. The local dendritic interactions indicated by the heterosynaptic spread of plasticity could involve (1) sharing of plasticity-related signaling molecules that spread from the active inputs to adjacent synapses, which might favor the clustering of postsynaptic strengths [46,51]; and (2) competition for resources and signaling molecules among asynchronously active inputs, which promote input specificity of the winners [49–51] (Fig 8, inset). According to such scenarios of local interactions, the conversion of depression to potentiation observed at distal spines during LTD in organotypic slices could be explained by the sequestering of “depression signals” by the stimulated spine and its near neighbors, and the slightly distant spines may experience the depletion of depression signals to trigger their de-repression. Notably, in contrast to the spatially confined spread of postsynaptic depression in organotypic slices, the depression was more widespread across the dendritic arbor in dissociated cultures (see also [91]). In a preparation that preserves the physiological circuit, the structured input pattern could help drive the organization of a compartmentalized signaling network along the dendritic shaft that permits for controlled sharing and competition for signals/resources [17,49]. Importantly, along with previous studies in organotypic slices or in vivo [29,51,92], our observations are in agreement with recent theoretical models that predict the spatial clustering of the strengths of synchronously active synapses that enhances the contrast with the strengths of inactive, more distant, synapses [30,93–95]. The heterosynaptic postsynaptic interactions may differentially influence presynaptic neurotransmitter release of stimulated versus nonstimulated synapses through retrograde signaling [35,60,96,97] and contribute to the dendritic spatial clustering of presynaptic strengths for boutons from the same axon.

A variety of retrograde mechanisms involving the release of diffusible cues [96,98] and direct signaling via synapse adhesion proteins [28,99,100] regulate presynaptic strength. The interactions along the dendrite between active and inactive spines, therefore, could contribute to the observed dendritic spatial clustering of presynaptic strengths of boutons from the same axon through differential retrograde signaling [35,60,97]. Given that spine competition is driven by the presynaptic activity in the first place, the retrograde regulation could serve to match the pre- and the postsynaptic strengths [66] to enhance input specificity [101] or provide a compensatory regulation to constrain local dendritic activity [35,97]. When and how the two opposing forms of retrograde regulation are engaged to control the release probability at the level of individual synapses remain to be clarified, although our plasticity experiments provide some insights (see below). Importantly, presynaptic strengths are also subject to feedback regulation by glial signaling [48,102,103], which underscores the complexity of synaptic strength tuning mechanisms.

The finding that the presynaptic cell has a major influence on the branchwise spatial distribution, especially of presynaptic strengths, is also reflected in the expression of synaptic plasticity. In both dissociated cultures and organotypic slices, the 1- to 3-Hz stimulation of presynaptic cells produces variable EPSC amplitude changes that are associated with changes in PPR at unitary connections and RRP size at individual boutons. The changes in PPR and RRP size, which are parameters related to release probability [7,104,105], suggest a presynaptic contribution to the plasticity. Interestingly, both measurements of PPR and RRP size suggest that plasticity induction results in the normalization of presynaptic strengths, whereby connections and/or synapses with low initial *p*_*r*_ show a large increase in *p*_*r*_ and vice versa. Such activity-dependent normalization of presynaptic strengths has been previously observed in dense synaptic network in dissociated cultured neurons [106] and following long-term plasticity induction in acute slices in hippocampal neurons and cortical layer 2/3 and layer 4 neurons [107,108]. The function of normalization of RRP size or *p*_*r*_ in presynaptic plasticity may represent the reduction in variance towards an optimization for reliable synaptic transmission that is proposed for a statistical form of long-term synaptic plasticity (see below) [109]. Mechanistically, the presynaptic change in RRP may involve vesicle exchange between the recycling pool and the resting pool [110] and/or the vesicle superpool shared between boutons [111,112].

Importantly, our data from either primary cultures or organotypic slices—in which the long-term presynaptic potentiation induced by the low-frequency stimulation protocols is compensated by the postsynaptic Ca^2+^ and NMDA receptor–dependent depression, which rapidly spreads to nonstimulated synapses—suggest that such plasticity may represent a possible homeostatic mechanism [97]. Indeed, the same low-frequency stimulation can induce an increase in presynaptic strength, as evidenced by the reduced PPR or the increased RRP size, and, in parallel, a decrease in postsynaptic strength, as evidenced by the measurements of aEPSC amplitudes, SEP-GluA2 signal intensity, or spine head volume. Although a PPR decrease can represent a postsynaptic mechanism involving enhanced anchoring and accumulation of desensitized AMPA receptors at the postsynaptic density [113–116], such an occurrence is unlikely in our experiments; we find a concurrent decrease in SEP-GluA2 signal intensity or spine head volume, which suggests a reduction in synaptic AMPA receptors rather than an increase. In addition, similarly to our present findings, presynaptic potentiation that simultaneously accompanies postsynaptic depression has been reported previously in cultured hippocampal neurons upon global induction of metabotropic glutamate receptor–mediated depression with 3,5-Dihydroxyphenylglycine (DHPG) [117] and in cortical and hippocampal networks in acute slices [108,118]. The concurrent expression of pre- and postsynaptic plasticity in the opposite direction provides experimental support for theoretical modeling studies predicting that Hebbian plasticity must be compensated by rapid homeostatic processes in order to maintain network stability [26,59,119].

What might be the physiological function of postsynaptic depression that occurs concurrently to presynaptic LTP, and what might be the implications of the apparent lack of input specificity of postsynaptic depression? A recent study has presented a theoretical framework for the expression of long-term synaptic plasticity in which pre- and postsynaptic changes occur to reduce the error in eliciting a desired/given postsynaptic response [109]. A key element of the model is the differential impact of pre- and postsynaptic changes on the postsynaptic response statistics; e.g., an increase in *p*_*r*_ will increase the reliability and the mean size of postsynaptic responses by directly promoting the efficient coupling of AP to neurotransmitter release, whereas a postsynaptic potentiation that increases the quantal amplitude by an increase in the number of functional postsynaptic receptors may not only increase the postsynaptic response but also increase its variability [109]. In this framework, the observed presynaptic LTP (i.e., a presumed increase in *p*_*r*_) and a parallel postsynaptic depression involve pre- and postsynaptic changes whereby both changes are expected to enhance the reliability of information transmission. The spread of postsynaptic depression may help discriminate the postsynaptic response to the potentiated synapse by reducing the background dendritic activity, especially if the presynaptic potentiation of *p*_*r*_ is small [101]. Furthermore, the spread of postsynaptic depression to neighboring spines could serve a compensatory role in preventing overexcitation of the dendritic branch and saturation of the learning capacity of the neuron [59]. In physiological terms, such postsynaptic depression that is not input specific may, e.g., contribute to consolidation of learned memories during slow wave activity in sleep, which involves reactivation of memory traces along with a global scaling down of synaptic strengths [121–124].

## Materials and methods

### Ethics statement

All animal experiments were performed in accordance with the institutional regulations and guidelines of RIKEN and the University of Bordeaux and approved by the RIKEN Animal Experiment Committee and by the Ethical Committee of Bordeaux CE50.

### Primary cell cultures and transfections

Hippocampal cultures were prepared from P0–P1 rats and plated at low density onto an astrocyte monolayer. The cultures were maintained as described previously [35]. For imaging postsynaptic strengths, neurons were transfected with plasmids encoding SEP-GluA1 or SEP-GluA2 (kindly provided by Dr. Mark von Zastrow and Dr. Jeremy Henley, respectively) at DIV 10 using a Ca^2+^ phosphate protocol [60]. For imaging presynaptic strengths, neurons were transfected with a plasmid encoding VGLUT1-pHluorin (kindly provided by Dr. Robert Edwards) at DIV 6 using Lipofectamine 2000 (Invitrogen, Carlsbad, CA, USA). Cultures were used for imaging/electrophysiology experiments at DIV 10–14.

### Organotypic hippocampal slice culture and transfection through whole-cell recordings

Organotypic hippocampal slice cultures were prepared as described from wild-type mice (C57Bl6/J strain). Animals were raised in the animal facility at the University of Bordeaux; they were handled and euthanized according to European ethical rules. Briefly, animals at postnatal day 5–8 were quickly decapitated and brains placed in ice-cold Gey’s balanced salt solution under sterile conditions. Hippocampi were dissected out, and coronal slices (350 μm) were cut using a McIlwain tissue chopper (Campden Instruments, Leicester, UK) and incubated at 35°C with serum-containing medium on Millicell culture inserts (CM, Millipore, Burlington, MA, USA). The medium was replaced every 2–3 days. After 18–25 days in culture, slices were transferred to an aCSF containing (in mM) 130 NaCl, 2.5 KCl, 2 CaCl_2_, 1 MgCl_2_, 10 D-glucose, and 10 HEPEs (pH 7.35, osmolarity adjusted to 300 mOsm). Whole-cell patch clamp recordings were performed from pairs of CA3 pyramidal cells to test for functional connectivity using glass patch pipettes filled with a solution containing (in mM) 130 K-gluconate, 10 HEPES, 7 KCl, 0.05 EGTA, 2 Na_2_ATP, 2 MgATP, and 0.5 NaGTP (pH 7.30, osmolarity adjusted to 290 mOsm). Plasmids encoding EGFP or tdTomato were added to the pipette solution (100 ng μl^-1^) for subsequent visualization of neurons. After 5–10 min, the patch pipettes were slowly retracted resulting in membrane resealing and the formation of an outside-out patch. Slices were then returned to the incubator on their original inserts for another 24–36 h to allow for the expression of both EGFP and tdTomato by the two cells. Micropipettes were pulled from 1-mm diameter borosilicate capillaries (Harvard Apparatus, Cambridge, MA, USA) with a vertical puller (Narishige, Tokyo, Japan).

### Electrophysiology on dissociated neurons

Whole-cell patch-clamp recordings were carried out from cultures placed on the stage of an Olympus IX71 inverted microscope or an Olympus BX51 upright microscope (Olympus, Tokyo, Japan) at room temperature and using Axopatch 200B and multiclamp amplifiers (Axon Instruments, Molecular Devices, San Jose, CA, USA). The recording chamber was continuously perfused with an aCSF containing (in mM) 130 NaCl, 2.5 KCl, 2.2 CaCl_2_, 1.5 MgCl_2_, 10 D-glucose, 10 HEPES, and 0.1 picrotoxin (pH 7.35, osmolarity adjusted to 290 mOsm). The micropipettes were made from borosilicate glass capillaries, with a resistance in the range of 3–5 MΩ. The intracellular solution contained (in mM) 100 K-gluconate, 17 KCl, 5 NaCl, 5 MgCl_2_, 10 HEPES, 0.5 EGTA, 4 ATPK_2_, and 0.5 GTPNa (pH 7.3, osmolarity adjusted to 280 mOsm). For some recordings, internal solution contained BAPTA (30 mM) to buffer intracellular Ca^2+^ in the postsynaptic neuron.

To assess connectivity among neurons in simultaneous multiple recording experiments, each neuron was stimulated by 1–2 ms step depolarization from -80 mV to +20 mV in V-clamp mode while identifying the neurons responding with EPSCs. Monosynaptic connections were identified by a short latency between the stimulation artefact and EPSC onset (<10 ms). EPSCs were sampled every 15 s by alternating the presynaptic stimulation between the convergent inputs. PPR was determined by delivering two stimuli 50 ms apart and dividing the peak response to the second stimulus by the peak response of the first one. Evoked aEPSCs were recorded in Sr^2+^-containing aCSF in which 2.2 mM CaCl_2_/1.5 mM MgCl_2_ was replaced with 1.5 mM Sr^2+^/2.2 mM MgCl_2_. The amplitude and frequency of quantal events were measured between 50 and 500 ms after the stimulation. Recorded events were filtered at 2 kHz and sampled at 10 kHz using the pClamp software (Axon Instruments). Amplitude and frequency of the quantal events were analyzed using Mini Analysis software (Synaptosoft, Decatur, GA, USA). The detection threshold was set at -5 pA. Cells with unstable baselines were discarded.

### Electrophysiology on organotypic slices

Whole-cell patch-clamp recordings were carried out from hippocampal organotypic slices placed on the stage of a Nikon Eclipse FN1 upright microscope (Nikon, Tokyo, Japan) at RT and using a Multiclamp 700B amplifier (Axon Instruments). The recording chamber was continuously perfused with ACSF bubbled with 95% O2/5% CO_2_ at room temperature and containing (in mM) 125 NaCl, 2.5 KCl, 26 NaHCO_3_, 1.25 NaH2PO_4_, 4 CaCl2, 4 MgCl_2_, and 25 glucose. The recording pipettes were filled with intracellular solution containing (in mM) 130 K-gluconate, 10 HEPES, 7 KCl, 0.05 EGTA, 2 Na_2_ATP, 2 MgATP, and 0.5 NaGTP (pH 7.30, osmolarity adjusted to 290 mOsm). CA3 pyramidal neurons were identified either with DIC or by visualizing the EGFP and tdTomato fluorescence. Unitary AMPA-receptor–mediated EPSCs were evoked every 10 s by injected 2–3 nA for 2 ms in the presynaptic neuron to elicit an AP in current-clamp mode while clamping the membrane potential of the postsynaptic neuron at -70 mv. The series resistance (Rs) was left uncompensated. Recordings with Rs higher than 30 MΩ were discarded. LTD was induced by eliciting APs at 3 Hz for 3 min in the presynaptic cell while keeping the postsynaptic cell under current clamp at RMP [83]. LTP was induced by stimulating the afferent Schaffer’s collaterals at 2 Hz for 100 s while maintaining the postsynaptic cell under voltage clamp at 0 mV [85]. Spontaneous synaptic currents were recorded in between stimulations of the presynaptic neuron. PPR was determined by delivering two pulses separated by 50 ms. PPR was measured from average traces and defined as the peak current of the second EPSC over the peak current of the first EPSC. EPSC amplitudes and PPR measurements were performed using Clampfit (Axon Instruments). EPSC kinetics were analyzed using Mini Analysis software (Synaptosoft).

### Live-cell imaging on cultured dissociated neurons

For measurement of the RRP using the FM4-64 dye, images were acquired on an inverted Olympus IX71 microscope equipped with a Micromax-cooled CCD camera (Princeton Instruments, Acton, MA, USA) driven by Metamorph software (Molecular Devices). After performing multiple patch clamp on 3–4 neurons and filling the postsynaptic neuron with an Alexa Fluor dye via the patch pipette, synapses were labeled with FM4-64 by stimulating the presynaptic neurons with 40 APs at 20 Hz in an aCSF containing 10 μM of FM4-64 dye, CNQX (20 μM), and APV (50 μM) at room temperature. Neurons were left in presence of FM4-64 for a further minute to allow completion of endocytosis and then washed in normal aCSF. Advasep-7 (1 mM; Biotium, Fremont, CA, USA) was included for the first minute of the washing procedure to assist with FM dye removal from membranes. After replacing with normal aCSF, images were acquired before and after FM dye unloading stimulation triggered by 600 APs at 10 Hz in the presynaptic cells. The remaining signal was taken as background.

For measurement of the RRP at single boutons using VGLUT1-pHluorin, the transfected neuron was patch clamped along with a postsynaptic neuron filled with 100 μM AF 594 dye. The presynaptic neuron was stimulated at 20 Hz for 2 s (100 mV, 1–2 ms step depolarization) under V-clamp. Time-lapse VGLUT1-pH images were acquired at 1Hz on an iXon EMCCD camera (Andor Technology, Belfast, UK) driven by Metamorph software (Molecular Devices). ΔF/F_0_ for identified active boutons was measured after subtracting local background, where F_0_ was the initial fluorescence. This measurement was repeated before and 20–30 min after the stimulation given to the presynaptic neuron (180 APs elicited at 1 Hz). For each cell, absolute values of differences in RRP size of each pair were normalized to the standard deviation of all pair comparisons.

To measure postsynaptic AMPAR abundance at single synapses, dual patch-clamp recordings were performed from a SEP-GluA1/2 transfected postsynaptic neuron along with a presynaptic partner filled with an AF350 or AF594 dye to visualize the axon and help identify putative synapses formed between the two neurons. SEP-GluA1 and SEP-GluA2 images were acquired on a Micromax-cooled CCD camera (Princeton Instruments) driven by Metamorph software (Molecular Devices). Images were collected every 20 s; the integrated fluorescence was measured after subtracting the local background for each identified receptor subunit cluster. Absolute values of pair differences in SEP-GluA fluorescence intensity were normalized to the standard deviation of all pair comparisons for a given cell.

### Confocal laser scanning microscopy and analysis of spine morphology on fixed organotypic hippocampal slices

For visualization of putative synaptic contacts between CA3 transfected cells, organotypic slices were fixed with 4% paraformaldehyde and 4% sucrose in PBS for 4 h, washed in PBS, and subsequently mounted in Mowiol. Images were acquired on a commercial Leica TCS SP8 microscope (Wetzlar, Germany) using a 63×/1.4 NA oil objective and a pinhole opened to 1× the Airy disk. Images of 2,048 × 2,048 pixels, corresponding to a pixel size of 80–85 nm, were acquired at a scanning frequency of 400 Hz. The vertical step size was set at 0.3 μm. Spine morphology was analyzed from 2D projections of confocal image stacks in ImageJ (NIH) [125] using a custom-written plugin, which involves the following steps. First, a binary representation of the original image is generated by a wavelet filtering algorithm [126], allowing identification of dendritic structures as individual objects. Because this process may separate spines from the dendritic shaft depending on the local fluorescence, the second step consists in the automated reconnection of those spines to the dendrite shaft by finding the optimal path between the two, following a gradient field computed on the original image [127]. At this stage, the binary image accurately captures the dendritic structure. From its outline, we compute a Delaunay triangulation used to generate a skeleton that topologically represents the dendrite and is used to properly segment the spine head, further fitted as an ellipse. From this ellipse, the major (*a*) and minor (*b*) axis are extracted, and the volume is estimated by giving the third orthogonal axis as the volume is computed as $\frac{4}{3}\pi ab^{2}$.

### Confocal spinning disk microscopy and analysis of spine morphology in live organotypic hippocampal slices

To monitor spine structural remodeling upon eliciting synaptic plasticity, organotypic slices were imaged in the same live conditions as for electrophysiological recordings with a spinning disk confocal unit CSU10 from Yokogawa (Tokyo, Japan) fed by a 4-color laser bench (Roper Scientific, Trenton, NJ, USA) and attached to an upright microscope equipped with a 60×/1.0 NA water immersion objective (Nikon Eclipse FN1). Stacks of confocal images in the GFP and tdTomato channels (laser lines 491 and 561, respectively), were acquired using a Rolera-em-c^2^ EMCCD camera from Qimaging (Surrey, BC, Canada) driven by Metamorph software (Molecular Devices). Acquired images contained 1,004 × 1,002 pixels with pixel size of 125 nm. The vertical step size was set at 0.3 μm and controlled using a PIFOC piezo objective z-scanner (Physik Instrumente, Karlsruhe, Germany) driven by Metamorph. We estimated the head volume of spines emanating laterally from the dendritic shaft on 2D projections of the confocal stacks. After subtracting the background fluorescence, we normalized the summed intensity pixel values in a 13 × 13 pixels area containing the spine head to the average intensity pixel value of the neighboring shaft. The resulting relative fluorescence intensity is expected to be proportional to the accessible spine head volume [128].

### Statistics

For normally distributed data (as determined by the d’Agostino–Pearson normality test), differences were tested using the paired or unpaired two-tailed Student *t* test or one-way ANOVA. The Mann–Whitney test, the Wilcoxon rank test, or the Kruskal–Wallis test were used when criteria for normality were not met. In figures, statistical significance is indicated by * for *p* < 0.05, ** for *p* < 0.01, *** for *p* < 0.001, and **** for *p* < 0.0001. GraphPad Prism software was used for statistical analysis. Data were expressed as mean ± SEM.

## Supporting information

S1 Fig Correlation of aEPSC amplitude and waveform between convergent inputs is not associated with changes in series resistance. Related to Fig 2. (A,B) Rise time and decay time constant values are correlated between the two inputs (rise time: R^2^ = 0.82, *p* = 0.0018, decay time constant: R^2^ = 0.77, *p* = 0.0042). Linear regression line and 95% confidence interval (gray shaded area) are shown. (C–E) aEPSC amplitude (C), rise-time values (D), and decay time constant (E) are not impacted by the differences in series resistance over the range measured during the triple recordings. Underlying data can be found in S1 Data. aEPSC, asynchronous EPSC; EPSC, excitatory postsynaptic current.
(TIF)

S2 Fig SEP-GluA1 and SEP-GluA2 fluorescence signal intensity as a proxy for postsynaptic strengths. Related to Fig 2. (A) Example traces of mEPSC recordings (left) and amplitude histogram of mEPSCs normalized to the median (right) and fitted by the lognormal function (black curve, R^2^ = 0.99). (B–C) Representative images of dendrites from neurons expressing SEP-GluA1 (B, left) or SEP-GluA2 (C, left) and corresponding histograms of normalized integrated intensity of SEP-GluA fluorescence puncta (right) fitted by the lognormal function (black curves: SEP-GluA1, R^2^ = 0.93; SEP-GluA2, R^2^ = 0.98) (D) Cumulative distributions of normalized mEPSC amplitudes and normalized signal intensity of individual SEP-GluA1 and SEP-GluA2 fluorescence puncta. Underlying data can be found in S1 Data. GluA, AMPA receptor subunit; EPSC, excitatory postsynaptic current; mEPSC, miniature EPSC; SEP, superecliptic pHluorin.
(TIF)

S3 Fig Changes in aEPSC frequency are not correlated between stimulated and nonstimulated inputs. Related to Fig 4. Comparison of the extent change in aEPSC frequency before and after the application of CS (1 Hz, 3 min) at stimulated versus nonstimulated synapses. Underlying data can be found in S1 Data. aEPSC, asynchronous EPSC; CS, conditioning stimulation; EPSC, excitatory postsynaptic current.
(TIF)

S4 Fig Uniform downscaling of postsynaptic AMPA receptors is calcium dependent. Related to Fig 4. (A) Representative traces showing mEPSC events before and after the CS recorded from a control neuron (black) or a neuron filled with 10 mM BAPTA (blue). (B) Plots of mEPSC amplitude before versus after the CS for control (left) and BAPTA-filled (right) neurons. Underlying data can be found in S1 Data. AMPA, α-amino-3-hydroxy-5-methyl-4-isoxazolepropionic acid; BAPTA, 1,2-bis(o-aminophenoxy)ethane-N,N,N′,N′-tetraacetic acid; CS, conditioning stimulation; EPSC, excitatory postsynaptic current; mEPSC, miniature EPSC.
(TIF)

S5 Fig Relationship between EPSC amplitude, PPR, and SEP-GluA2 signal intensity changes. Related to Fig 5. Individual recordings showing that LTD of EPSC amplitude (left column) (A,B) is associated with a decrease in SEP-GluA2 fluorescence intensity (right column), whereas LTP of EPSC amplitude (C) is associated with a decrease in PPR (middle column). Opposite changes in PPR and SEP-GluA2 fluorescence intensity (D) are associated with no net change in EPSC amplitude. (E, F) Comparison of EPSC amplitude change versus PPR change (E) and SEP-GluA2 fluorescence change (F). Underlying data can be found in S1 Data. EPSC, excitatory postsynaptic current; GluA, AMPA receptor subunit; LTD, long-term depression; LTP, long-term potentiation; PPR, paired-pulse ratio; SEP, superecliptic pHluorin.
(TIF)

S6 Fig Electrophysiological properties of unitary EPSCs at CA3–CA3 recurrent connections. Related to Fig 6. (A) Summary of EPSC amplitude, PPR, peak EPSC amplitude excluding failures, rise time, decay time constant, and latency at the time of transfection through whole-cell recordings in CA3 neurons. (B) Left: plot showing average maximal EPSC values (peak EPSC amplitude excluding failures) during the first and second patch-clamp recording sessions (*n* = 10 CA3–CA3 pairs, Wilcoxon matched pairs signed-rank test). Right three panels: the transfection procedure does not produce consistent changes in rise time, decay time constant, and latency of EPSCs (*n* = 10 pairs, Wilcoxon matched pairs signed-rank test). Underlying data can be found in S1 Data. CA3, Cornu Ammonis 3; EPSC, excitatory postsynaptic current; PPR, paired-pulse ratio.
(TIF)

S7 Fig LTD induction is associated with a consistent change in PPR, whereas LTP induction is not accompanied by consistent changes in PPR nor sEPSC amplitude at unitary CA3 recurrent connections. Related to Fig 7. (A) Comparison of the PPR change versus initial PPR (PPR_0_) for LTD experiments in absence or presence of D-AP5 (LTD [without D-AP5]: R^2^ = 0.57, *p* = 0.0115). (B) Comparison of the PPR change versus EPSC amplitude change for LTD experiments in absence of D-AP5 (R^2^ = 0.77, *p* = 0.0042). Linear regression and 95% confidence interval (gray) are shown. (C) Left, representative traces showing synaptic currents in the postsynaptic CA3 neuron evoked by a pair of APs triggered in the presynaptic CA3 neuron (2–3 nA, 50-ms interval), before and 20 min after LTP induction. Right, summary of the time course of EPSC amplitude (*n* = 8 cell pairs). The gray shaded box represents the LTP induction. (D) Left, plot showing PPR values before and after LTP induction (*n* = 10 cell pairs, Wilcoxon matched pairs signed-rank test). Right, comparison of the PPR change versus initial PPR (PPR_0_) (R^2^ = 0.77, *p* = 0.0096). Linear regression and 95% confidence interval (gray) are shown. (E) Left, example traces of sEPSCs recorded from a postsynaptic CA3 neuron before (Pre-stim) and after (Post-stim) LTP induction. Middle, plot showing sEPSC amplitude before and after LTP induction (untreated: *n* = 7 cell pairs, Wilcoxon matched pairs signed-rank test). Right, cumulative distributions of sEPSC amplitudes before and after LTP induction. Underlying data can be found in S1 Data. AP, action potential; CA3, Cornu Ammonis 3; D-AP5, D-2-amino-5-phosphonovalerate; EPSC, excitatory postsynaptic current; LTD, long-term depression; LTP, long-term potentiation; PPR, paired-pulse ratio; sEPSC, spontaneous EPSC; stim, stimulation.
(TIF)

S1 Video Sequential unloading of the FM dye for the two inputs. Related to Fig 1. Images were obtained every 1 sec. After a baseline of 5 consecutive planes, 600 APs were delivered in the presynaptic cells at 10 Hz, thus triggering the loss of FM fluorescence at boutons corresponding to input 1 (left) or input 2 (right). AP, action potential.
(AVI)

S2 Video Imaging synaptic vesicle dynamics along the axon using VGLUT1-pH. Related to Fig 3. Time-lapse images were obtained before (baseline) or after (20 min) delivering the CS (180 APs at 1 Hz). Consecutive planes were obtained every 1 sec. After 15 consecutive planes, 40 APs (left and middle videos) or 600 APs (right video) were delivered at 20 Hz to mobilize the readily releasable or the total pool of presynaptic vesicles. AP, action potential; CS, conditioning stimulation; pH, pHluorin; VGLUT1, vesicular glutamate transporter 1.
(AVI)

S1 Data Data underlying figures and Supporting Information figures. Data for Fig 1C, 1D and 1H; Fig 2C–2E and 2H; Fig 3E–3H; Fig 4C–4L; Fig 5C, 5D and 5I–5N; Fig 6D and 6G; Fig 7A–7D and 7G; S1A–S1E Fig; S2A–S2D Fig; S3 Fig; S4B Fig; S5A–S5F Fig; S6A and S6B Fig; and S7A–S7E Fig.
(XLSX)

## Abbreviations

aCSF: artificial cerebrospinal fluid
aEPSC: asynchronous EPSC
AMPA: α-amino-3-hydroxy-5-methyl-4-isoxazolepropionic acid
AP: action potential
a.u: arbitrary unit
BAPTA: 1,2-bis(o-: amino: phenoxy: ): ethane: -N,N,N′,N′-tetra: acetic acid
CA3: Cornu Ammonis 3
CV: coefficient of variation
DG: dentate gyrus
DHPG: 3,5-Dihydroxyphenylglycine
DIV: days in vitro
D-AP5: D-2-amino-5-phosphonovalerate
EPSC: excitatory postsynaptic current
GFP: green fluorescent protein
GluA: AMPA receptor subunit
LTD: long-term depression
LTP: long-term potentiation
mEPSC: miniature EPSC
NMDA: N-methyl-D-aspartate
PPR: paired-pulse ratio
p_r_: neurotransmitter release probability
Ri: input resistance
RMP: resting membrane potential
RRP: readily releasable pool
Rs: series resistance
SEP: superecliptic pHluorin
sEPSC: spontaneous EPSC
VGLUT1: vesicular glutamate transporter 1
