# Supplementary figures and images for: Differential role of pre- and postsynaptic neurons in the activity-dependent control of synaptic strengths across dendrites
Source: PLoS Biol. 2019 Jun 5;17(6):e2006223. doi: 10.1371/journal.pbio.2006223 (PMC6576792; doi:10.1371/journal.pbio.2006223)

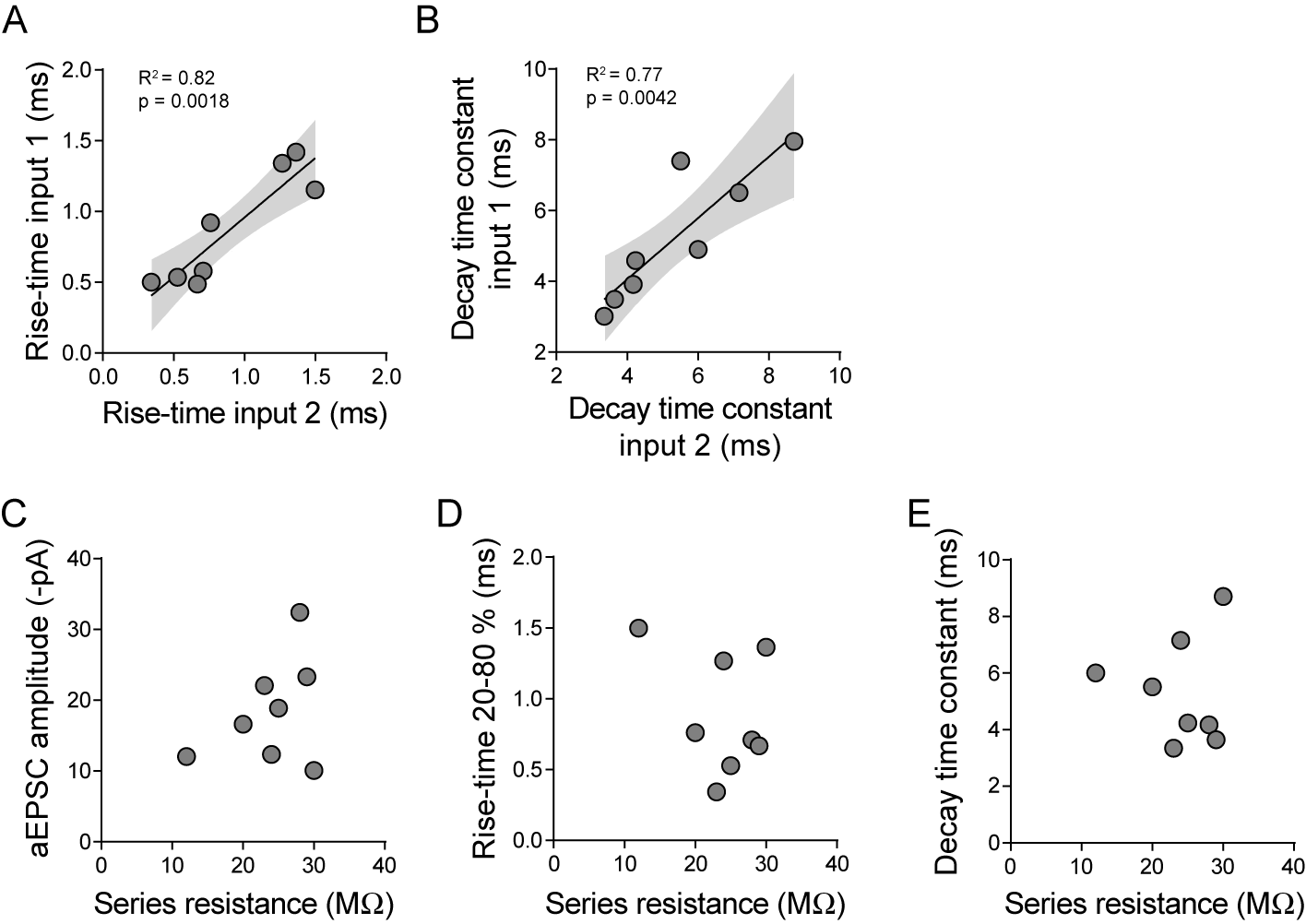

Supplement: S1 Fig — Related to Fig 2. (A,B) Rise time and decay time constant values are correlated between the two inputs (rise time: R2 = 0.82, p = 0.0018, decay time constant: R2 = 0.77, p = 0.0042). Linear regression line and 95% confidence interval (gray shaded area) are shown. (C–E) aEPSC amplitude (C), rise-time values (D), and decay time constant (E) are not impacted by the differences in series resistance over the range measured during the triple recordings. Underlying data can be found in S1 Data. aEPSC, asynchronous EPSC; EPSC, excitatory postsynaptic current. (TIF) [file pbio.2006223.s001.tif]

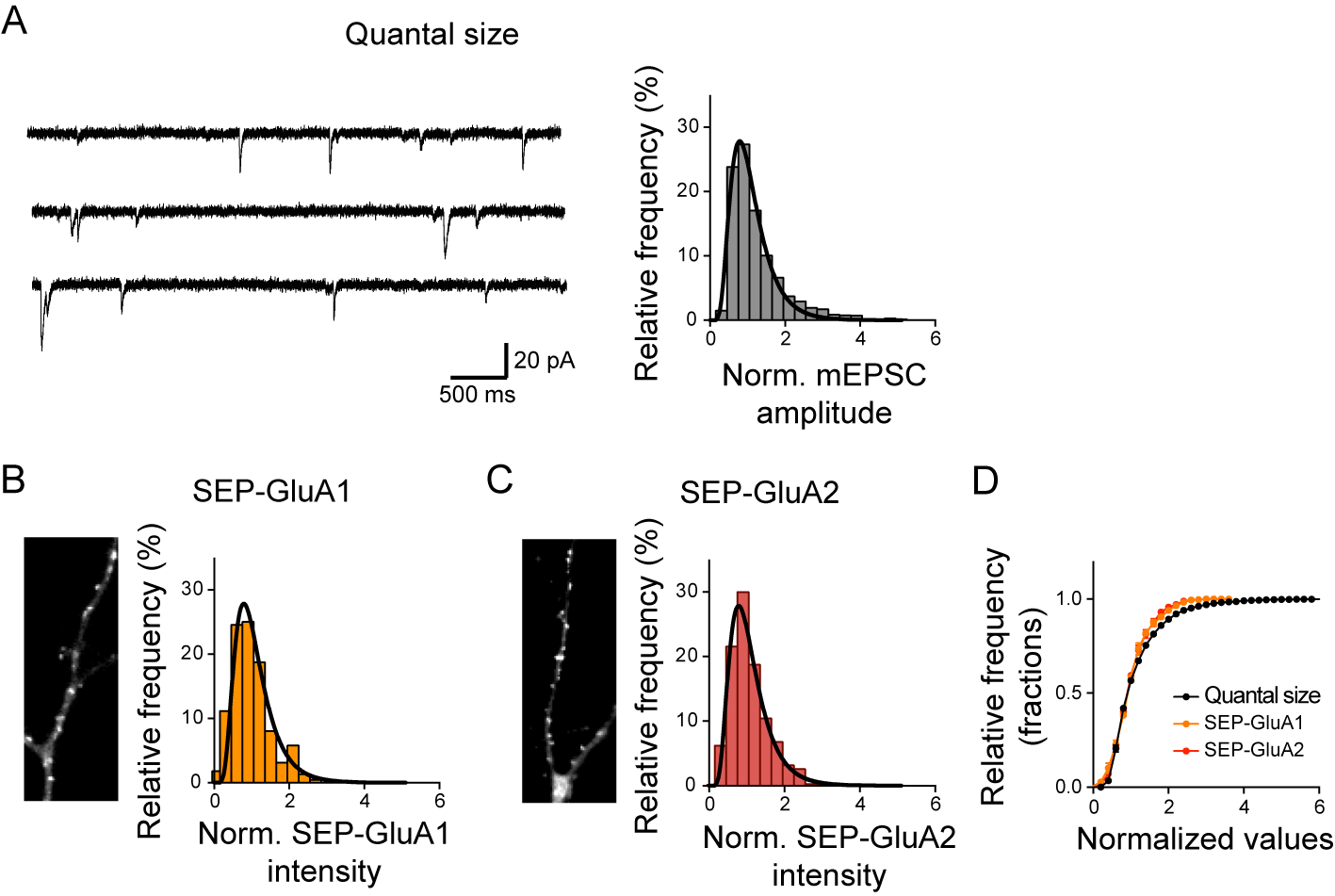

Supplement: S2 Fig — Related to Fig 2. (A) Example traces of mEPSC recordings (left) and amplitude histogram of mEPSCs normalized to the median (right) and fitted by the lognormal function (black curve, R2 = 0.99). (B–C) Representative images of dendrites from neurons expressing SEP-GluA1 (B, left) or SEP-GluA2 (C, left) and corresponding histograms of normalized integrated intensity of SEP-GluA fluorescence puncta (right) fitted by the lognormal function (black curves: SEP-GluA1, R2 = 0.93; SEP-GluA2, R2 = 0.98) (D) Cumulative distributions of normalized mEPSC amplitudes and normalized signal intensity of individual SEP-GluA1 and SEP-GluA2 fluorescence puncta. Underlying data can be found in S1 Data. GluA, AMPA receptor subunit; EPSC, excitatory postsynaptic current; mEPSC, miniature EPSC; SEP, superecliptic pHluorin. (TIF) [file pbio.2006223.s002.tif]

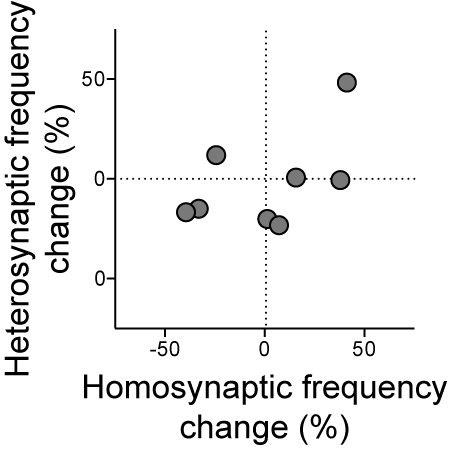

Supplement: S3 Fig — Related to Fig 4. Comparison of the extent change in aEPSC frequency before and after the application of CS (1 Hz, 3 min) at stimulated versus nonstimulated synapses. Underlying data can be found in S1 Data. aEPSC, asynchronous EPSC; CS, conditioning stimulation; EPSC, excitatory postsynaptic current. (TIF) [file pbio.2006223.s003.tif]

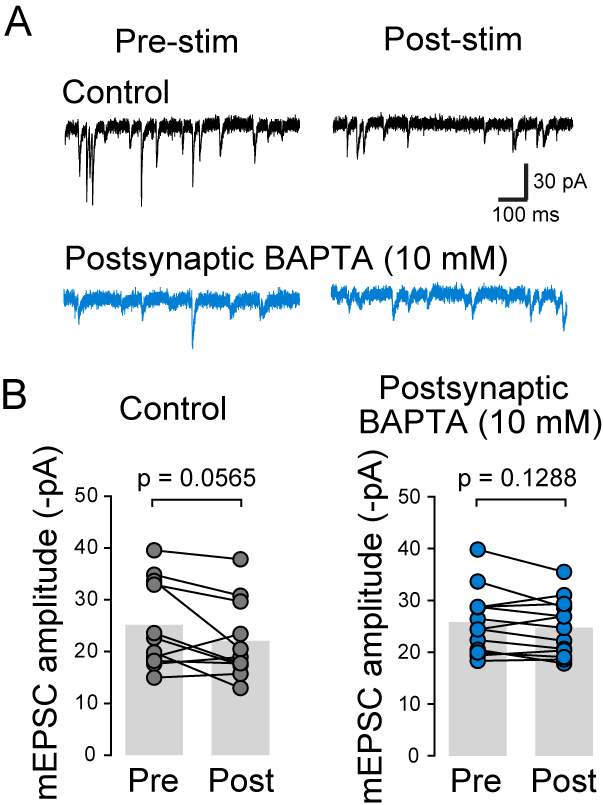

Supplement: S4 Fig — Related to Fig 4. (A) Representative traces showing mEPSC events before and after the CS recorded from a control neuron (black) or a neuron filled with 10 mM BAPTA (blue). (B) Plots of mEPSC amplitude before versus after the CS for control (left) and BAPTA-filled (right) neurons. Underlying data can be found in S1 Data. AMPA, α-amino-3-hydroxy-5-methyl-4-isoxazolepropionic acid; BAPTA, 1,2-bis(o-aminophenoxy)ethane-N,N,N′,N′-tetraacetic acid; CS, conditioning stimulation; EPSC, excitatory postsynaptic current; mEPSC, miniature EPSC. (TIF) [file pbio.2006223.s004.tif]

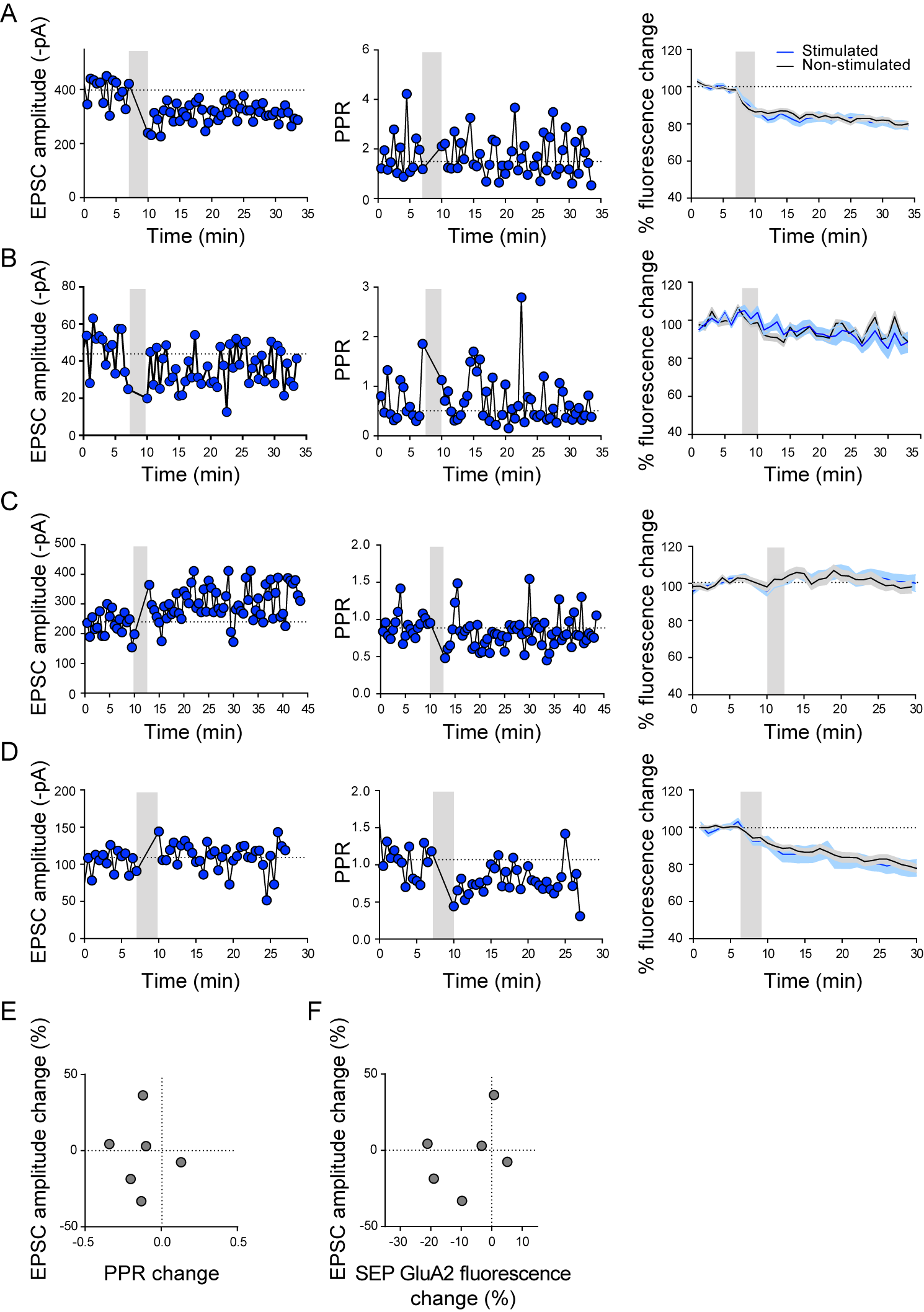

Supplement: S5 Fig — Related to Fig 5. Individual recordings showing that LTD of EPSC amplitude (left column) (A,B) is associated with a decrease in SEP-GluA2 fluorescence intensity (right column), whereas LTP of EPSC amplitude (C) is associated with a decrease in PPR (middle column). Opposite changes in PPR and SEP-GluA2 fluorescence intensity (D) are associated with no net change in EPSC amplitude. (E, F) Comparison of EPSC amplitude change versus PPR change (E) and SEP-GluA2 fluorescence change (F). Underlying data can be found in S1 Data. EPSC, excitatory postsynaptic current; GluA, AMPA receptor subunit; LTD, long-term depression; LTP, long-term potentiation; PPR, paired-pulse ratio; SEP, superecliptic pHluorin. (TIF) [file pbio.2006223.s005.tif]

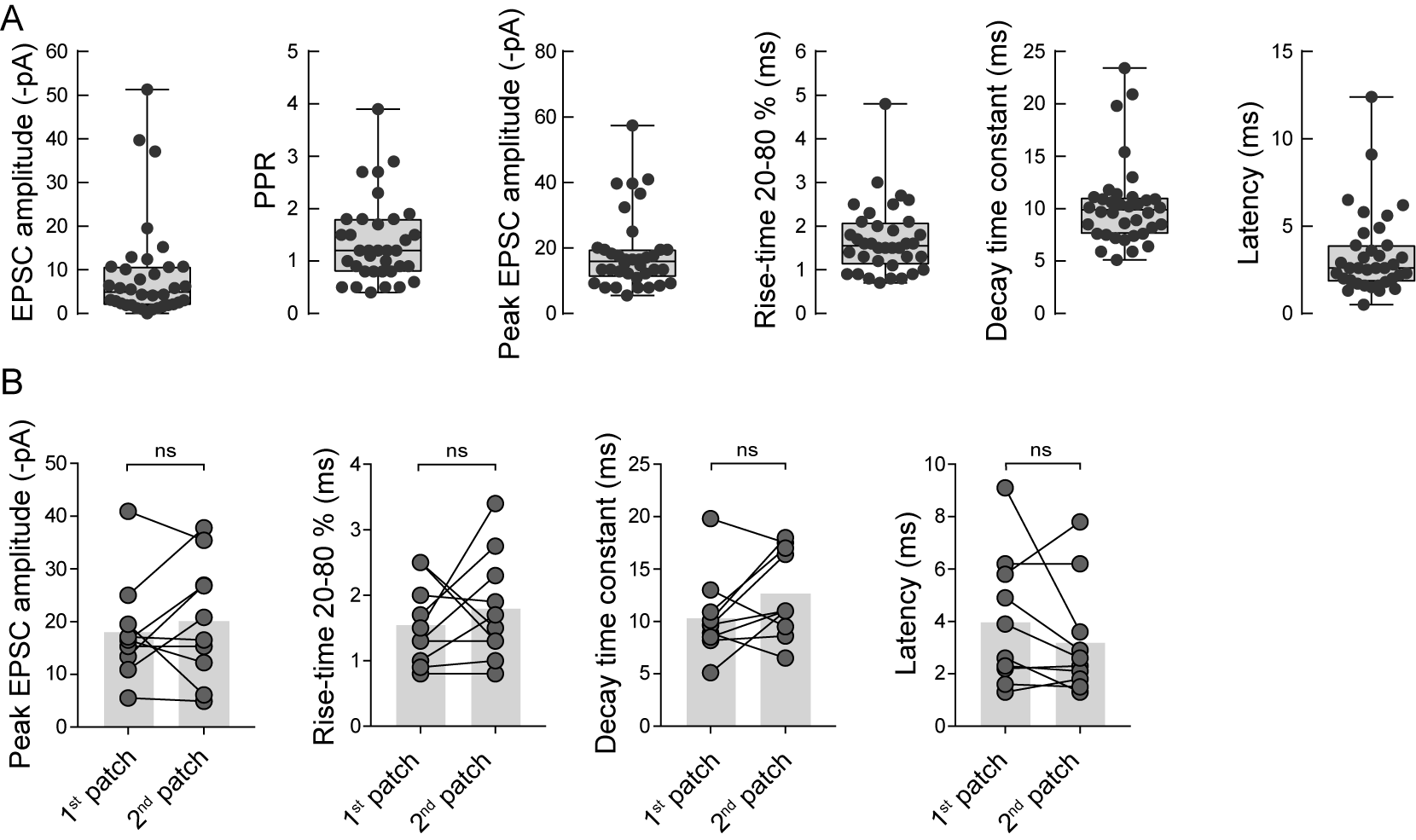

Supplement: S6 Fig — Related to Fig 6. (A) Summary of EPSC amplitude, PPR, peak EPSC amplitude excluding failures, rise time, decay time constant, and latency at the time of transfection through whole-cell recordings in CA3 neurons. (B) Left: plot showing average maximal EPSC values (peak EPSC amplitude excluding failures) during the first and second patch-clamp recording sessions (n = 10 CA3–CA3 pairs, Wilcoxon matched pairs signed-rank test). Right three panels: the transfection procedure does not produce consistent changes in rise time, decay time constant, and latency of EPSCs (n = 10 pairs, Wilcoxon matched pairs signed-rank test). Underlying data can be found in S1 Data. CA3, Cornu Ammonis 3; EPSC, excitatory postsynaptic current; PPR, paired-pulse ratio. (TIF) [file pbio.2006223.s006.tif]

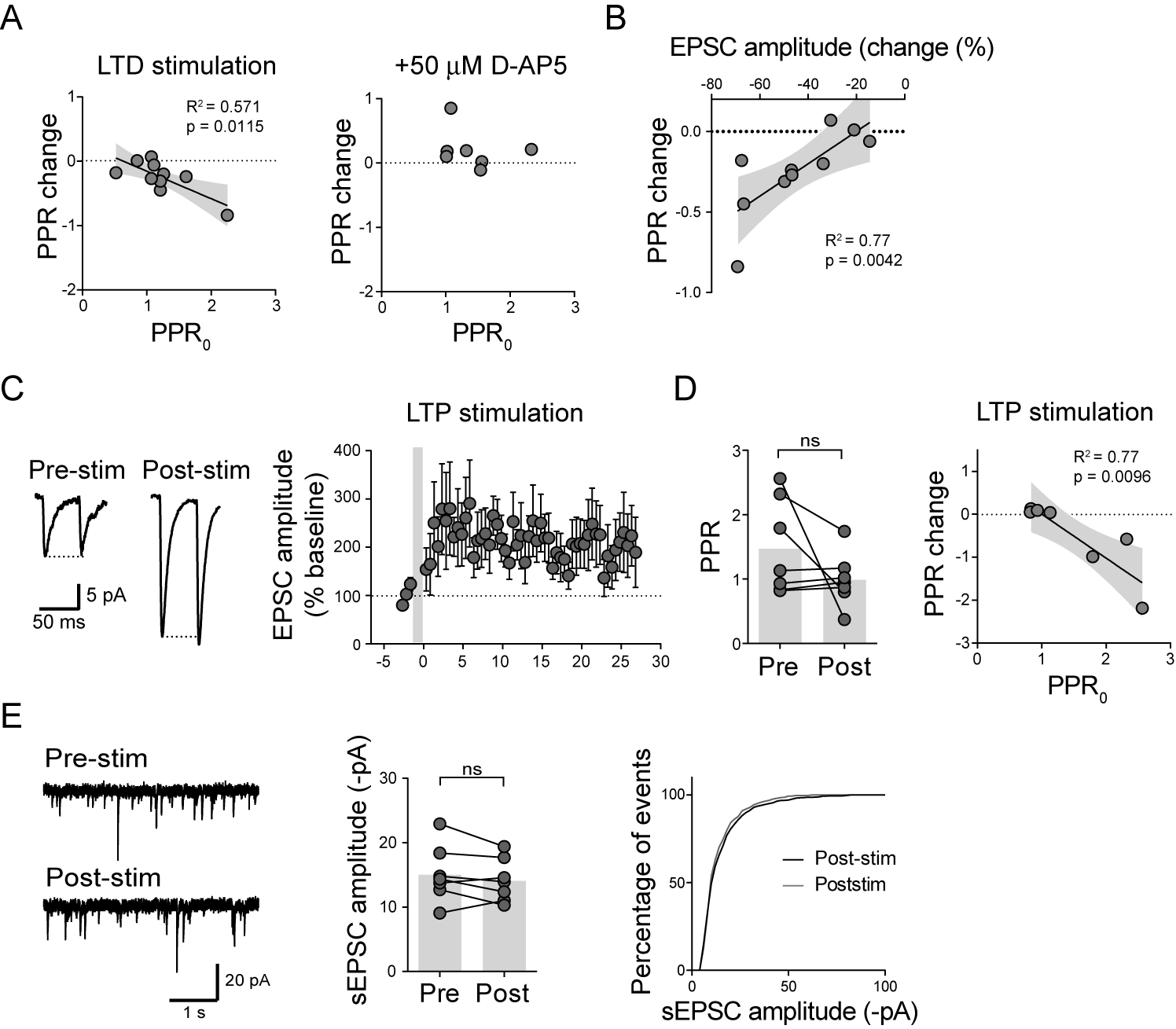

Supplement: S7 Fig — Related to Fig 7. (A) Comparison of the PPR change versus initial PPR (PPR0) for LTD experiments in absence or presence of D-AP5 (LTD [without D-AP5]: R2 = 0.57, p = 0.0115). (B) Comparison of the PPR change versus EPSC amplitude change for LTD experiments in absence of D-AP5 (R2 = 0.77, p = 0.0042). Linear regression and 95% confidence interval (gray) are shown. (C) Left, representative traces showing synaptic currents in the postsynaptic CA3 neuron evoked by a pair of APs triggered in the presynaptic CA3 neuron (2–3 nA, 50-ms interval), before and 20 min after LTP induction. Right, summary of the time course of EPSC amplitude (n = 8 cell pairs). The gray shaded box represents the LTP induction. (D) Left, plot showing PPR values before and after LTP induction (n = 10 cell pairs, Wilcoxon matched pairs signed-rank test). Right, comparison of the PPR change versus initial PPR (PPR0) (R2 = 0.77, p = 0.0096). Linear regression and 95% confidence interval (gray) are shown. (E) Left, example traces of sEPSCs recorded from a postsynaptic CA3 neuron before (Pre-stim) and after (Post-stim) LTP induction. Middle, plot showing sEPSC amplitude before and after LTP induction (untreated: n = 7 cell pairs, Wilcoxon matched pairs signed-rank test). Right, cumulative distributions of sEPSC amplitudes before and after LTP induction. Underlying data can be found in S1 Data. AP, action potential; CA3, Cornu Ammonis 3; D-AP5, D-2-amino-5-phosphonovalerate; EPSC, excitatory postsynaptic current; LTD, long-term depression; LTP, long-term potentiation; PPR, paired-pulse ratio; sEPSC, spontaneous EPSC; stim, stimulation. (TIF) [file pbio.2006223.s007.tif]
